# Comparative efficacy of inhalers in mild-to-moderate asthma: systematic review and network meta-analysis

**DOI:** 10.1038/s41598-022-09941-z

**Published:** 2022-04-08

**Authors:** Hyung Jun Park, Jin-Young Huh, Ji Sung Lee, Jae Seung Lee, Yeon-Mok Oh, Sei Won Lee

**Affiliations:** 1 Department of Pulmonary and Critical Care Medicine, Asan Medical Center, University of Ulsan College of Medicine, 88, Olympic-ro 43-gil, Songpa-gu, Seoul, 05505 South Korea; 2 Department of Clinical Epidemiology and Biostatistics, Clinical Research Center, Asan Medical Center, Seoul, South Korea

**Keywords:** Diseases, Medical research, Signs and symptoms

## Abstract

The comparative effectiveness of different inhaler therapies in mild-to-moderate asthma remains unclear. To assess this, we performed a systematic review and network meta-analysis of randomized controlled trials on the use of inhalers for mild-to-moderate asthma by searching PubMed, Cochrane, and Embase. A total of 29 trials including 43,515 patients and 12 types of inhaler therapies were included. For the prevention of severe and moderate-to-severe exacerbations, inhaled corticosteroid (ICS)/long-acting β2-agonist (LABA) as maintenance and reliever (SMART) showed the highest rank for effectiveness. As-needed ICS/LABA or short-acting β2-agonist (SABA) was similar to low-dose ICS and superior to as-needed SABA or LABA for the prevention of severe and moderate-severe exacerbations. As for lung function (FEV_1_), low-dose ICS/LABA had the highest rank; as-needed ICS/LABA was inferior to regular low-dose ICS but superior to placebo. Higher-dose ICS had a superior effect on the Asthma Control Questionnaire (ACQ) scores, and as-needed ICS/LABA and as-needed SABA or LABA had lower ranks in p-rankogram than did the regular use of low-dose ICS. As-needed ICS with LABA or SABA was more effective than a similar dose of regular ICS for preventing exacerbation in mild-to-moderate asthma. As-needed ICS showed some weakness in improving lung function and controlling asthma symptoms.

## Introduction

Asthma is a common and potentially serious chronic disease that imposes a substantial burden on patients by causing respiratory symptoms, limitation of activity, and exacerbations^1^, even with mild degrees of disease^2^. Until 2018, the Global Initiative for Asthma (GINA) guideline recommended that mild asthma may be controlled with either reliever medications (short-acting β_2_-agonists [SABA]) or low-dose inhaled corticosteroid (ICS)^3^. However, the GINA guideline published in 2019 states that as-needed SABA alone is no longer recommended due to safety issues^1^. Several randomized studies showed that SABA-only treatment does not significantly prevent severe exacerbations, and its frequent usage can actually increase the risk of severe exacerbations^1^. A recent study reported that as-needed ICS with rapid-acting long-acting β_2_-agonist (LABA) results in superior outcomes compared with as-needed SABA; interestingly, as-needed ICS with LABA showed non-inferior outcomes compared with regular ICS in the reduction of exacerbation^4–7^. Based on these studies, there have been recent changes in the treatment strategies for mild asthma^1^.

Several meta-analyses were performed to compare the effectiveness of different types of inhalers in asthma^8–11^. As-needed ICS/LABA without maintenance controller was inferior to regular low-dose ICS in a meta-analysis until 2017, although as-needed ICS/LABA was superior to as-needed SABA^12^. However, recent large-sized, randomized controlled trials (RCTs) showed that as-needed ICS/LABA is non-inferior or even superior to regular ICS for the prevention of severe exacerbations^5–7^, and a meta-analysis of that showed the effectiveness over regular ICS^13^. To overcome the limitations of single comparison of meta-analysis, two network meta-analyses were conducted to compare the effectiveness of asthma treatment strategies^14^ including as-needed ICS/LABA^15^. In these analyses, the regular use of higher-dose ICS or ICS/LABA seemed to be more effective than as-needed SABA for preventing exacerbations^14,15^. However, one study did not consider the asthma severity^14^, which may lead to the misunderstanding that strong inhalers are always more potent even in mild asthma. Another recent network meta-analysis did not include a number of relevant studies because the inclusion criteria only specified studies the direct comparison with as-needed ICS/LABA or SMART^15^.

Considering the limited amount of evidence on the comparison of inhalers in mild-to-moderate asthma, this systematic review compared the efficacies of a variety of inhalers in terms of exacerbation, lung function, and asthma control in patients with mild-to-moderate asthma. Based on this analysis, we aim to contribute to the development of a tailored treatment strategy in such patients according to their symptoms, lung function, and the risk for exacerbation.

## Methods

### Search strategy and study selection

The asthma severity was limited to mild-to-moderate cases to meet the transitivity, the methodological requirements of a network meta-analysis. We conducted a bibliographic search using PubMed, Embase, and Cochrane databases to find relevant studies published between January 2014 and March 2020. Moreover, we updated the search to January 2022. The previous studies published before 2014 were included from the database articles of previous meta-analyses^12,14,15^. As the meta-analyses were well conducted and the inclusion criteria were broader than our inclusion criteria, we assumed that it could be reasonable to replace the searching database before 2014. This systematic review was registered in PROSPERO (CRD42020209123).

The following eligibility criteria were used to specifically include patients with mild-to-moderate asthma. The eligible studies were RCTs that examined the long-term effects (> 24 weeks) of various inhaler strategies. Patients were aged > 5 years. Inhaler type was not restricted, but studies that compared dose differences smaller than the ICS dose category of the GINA guidelines were excluded. Mild-to-moderate asthma was defined as the baseline ICS dose of below the medium dose^1^ and mean forced expiratory volume in 1 s (FEV_1_) of above 75% of predicted or and Asthma Control Questionnaire (ACQ) score of < 1.5 if FEV_1_ was not presented. The study outcomes included at least one of the following: exacerbations of asthma, changes in FEV_1_, and ACQ score. Detailed search strategies are presented in the Supplementary Information. The latest search was conducted in January 2022.

### Data extraction

For each eligible study, two reviewers (HJP and JYH) independently selected the studies and abstracted the following items: year published, duration of follow-up, severity of asthma population judged by the authors, pretreatment and posttreatment of the values of FEV_1_ and the ACQ scores, number of patients who had a moderate or severe exacerbation, mean age, sample size, descriptions of interventions and comparators, treatment category, and definitions of exacerbation. Any discrepancies were resolved through a consensus with a third reviewer (SWL).

### Measuring treatment effect

According to the previously recommended definition for asthma outcomes^16^, severe exacerbations were defined as hospital admission, visit to an emergency department, or prescription of systemic corticosteroids for at least 3 days. Moderate exacerbations were defined as a decrease in pulmonary function (decrease in peak expiratory flow by > 20% from baseline), increase in the use of rescue drugs to > 8 puffs/day, any period of using systemic steroid or nighttime waking because of asthma symptoms (all for at least 2 consecutive days), and unscheduled visit to the clinic not requiring systemic steroids. Events meeting the criteria of both severe and moderate exacerbations were included in both categories. Although different versions of ACQ the (i.e., ACQ-5, ACQ-6, and ACQ-7) were used, all of them commonly have the information used in ACQ-5, while ACQ-6 additionally includes the use of rescue inhaler and ACQ-7 includes the predicted FEV_1_ values. The correlation among the different versions of ACQ had been validated in a previous study^17^.

### Risk of bias assessment

Two reviewers independently assessed the risk of bias using RoB2, a revised Cochrane risk-of-bias tool for randomized trials^18^. The tool included the response option of “definitely or probably yes” (assigned as a low risk of bias) and “definitely or probably no” (assigned as a high risk of bias). The items consisted of five components: randomization process, deviation from intended interventions, missing outcomes data, measurement of the outcome, and selection of the reported result.

### Statistical analysis

The frequentist network meta-analysis of aggregate data was performed to obtain the network estimates, and this article follows the PRISMA extension statement for network meta-analysis guideline. For the heterogeneity among studies, the random-effects model was used as a framework to calculate the comparison effect. For transitivity assumption, the comparability among treatment arms based on patient characteristics was explored to assess whether the characteristics were sufficiently similar. The intervention’s rank was calculated by P-score based on the point estimates and standard errors of the frequentist network meta-analysis estimates under normality assumption^19^. Heterogeneity was assessed with the Q statics, and the total inconsistency was calculated from the full design-by-treatment interaction random-effects model^20^. Additionally, the incoherence assumption (i.e., statistical disagreement between direct and indirect evidence in a closed loop) was evaluated by net splitting, also known as node splitting, and displayed as a forest plot. A comparison-adjusted funnel plot was used to evaluate the risk of publication bias^21^.

The metrics of the analysis for the primary outcome were as follows: exacerbations (effect measure, odds ratio), FEV_1_ change (effect measure, mean absolute difference), and ACQ change (effect measure, mean absolute difference). The exacerbation rate was calculated as the number of patients with exacerbations per patient-year. Because only summarized data can be assessed in most studies, we calculated the patient-year using the number of patients at randomization and at the end of the study period, and patients excluded at the end of the period were assumed as observed for half of the study period. All statistical analyses were performed using “netmeta” package of R software version 4.0.4 (R Foundation for Statistical Computing, Vienna, Austria).

## Results

### Data selection

**Figure 1:**
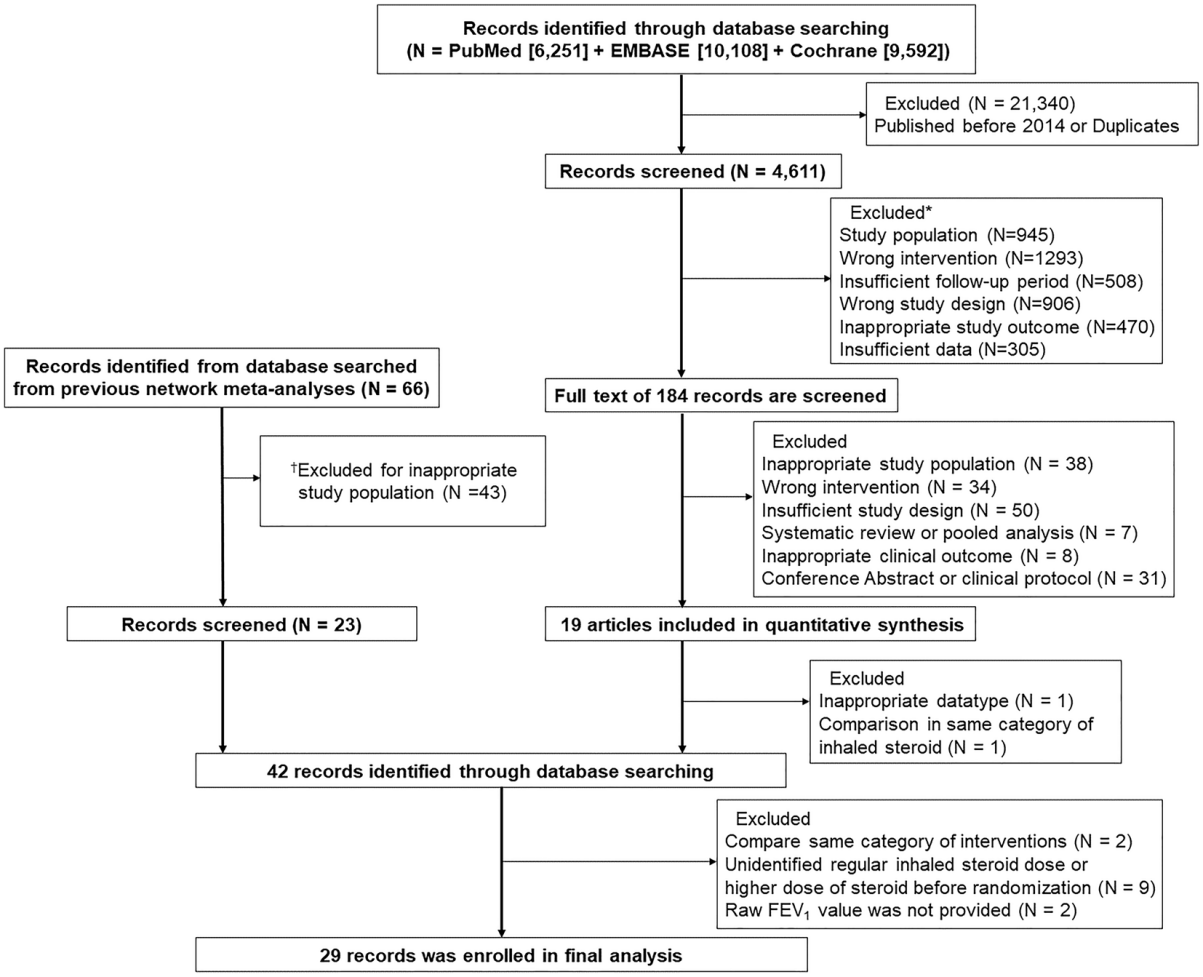
Study selection process. Asterisk: Records excluded in the screening of title, abstract. Dagger: Records excluded based on study population (mild and moderate asthma).

The systematic literature search identified 2661 relevant studies published between 2014 and 2022. After reviewing the study population, outcome, population, design, intervention, and follow-up period, 184 studies were selected for full-text review, through which 19 studies were considered to meet the inclusion criteria. Among the 66 studies published before 2014, 43 were excluded because of inappropriate study population. In the final review, 13 studies were excluded due to the following reasons: same category interventions^22,23^ unidentified regular controller’s steroid dose or high-dose ICS before randomization^24^, and no available FEV_1_ value^25,26^ (Fig. 1). Through this process, 29 studies with 43,515 patients and 12 different types of inhaler therapies were included in the final analysis (Supplementary Information).

### Study characteristics

In most of the study arms in the 29 studies, the mean age of the patients ranged between 30 and 40 years. Three studies were conducted in adolescents^27–29^. The mean predicted FEV_1_ and ACQ score of the studies were 88.2% and 1.33, respectively. The study sample size ranged from 44 to 11,693 patients, and the year of publication ranged from 1999 to 2020 (Supplementary Information). The numbers of studies with available data per outcome were 24 for exacerbations, 16 for FEV_1_ change, and 11 for ACQ.

### Classification of inhaler therapies

**Figure 2:**
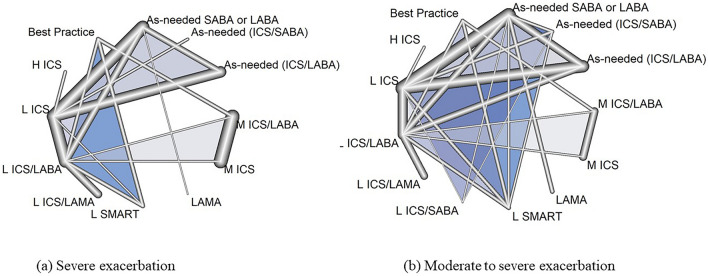
Network of inhaler therapies on the outcomes. The thickness of the network lines indicates the number of comparisons between each inhaler therapy. *H ICS* high-dose ICS, *M ICS* medium-dose ICS, *L ICS* low-dose ICS, *L ICS/LABA* low-dose ICS dose with LABA as maintenance, *M ICS/LABA* medium-dose ICS with LABA as maintenance, *L SMART* low-dose ICS with LABA as maintenance and reliever, *L ICS/LAMA* low-dose ICS with LAMA as maintenance, *L ICS/SABA* low-dose ICS with SABA as maintenance; best practice, adjustable dose of inhaler based on symptoms.

A total of 12 inhaler therapies were analyzed, and the inhalers and their doses are described in Supplementary Information. As-needed ICS/LABA or ICS/SABA was defined as no regular controller with the intermittent use of ICS with LABA or SABA^6,30^. Other classes were sorted based on the inhaled steroid dose (i.e., low-, medium-, and high-dose ICS) according to the equivalent dose based on the GINA guideline^1^. SMART was defined as the use of ICS/LABA as both maintenance and reliever therapies^1^. To clarify the difference in the maintenance ICS dose of SMART from regular medium-dose ICS as a controller, SMART was written as low-dose ICS of SMART (L SMART). Best practice was defined as the adjustable dose of a controller by physicians based on the patients’ symptoms. The placebo groups were patients who used placebo inhalers with an as-needed bronchodilator (SABA or LABA) that did not contain regular inhalers for symptom control. The networks of inhaler therapies according to the degree of exacerbation are shown in Fig. 2. The network shows the overall structure of comparisons among inhalers, which enable proper analysis with direct and indirect comparisons.

### Acute exacerbations of asthma according to inhaler therapy

A total of 21 trials including 39,915 patients were analyzed for severe exacerbations, and 24 trials including 41,741 patients were analyzed for moderate-to-severe exacerbations. Two studies were excluded from the severe exacerbation analysis because one study had no patients with severe exacerbation in the treatment arms^30^ and the other had not analyzed severe exacerbation^31^.

**Figure 3:**
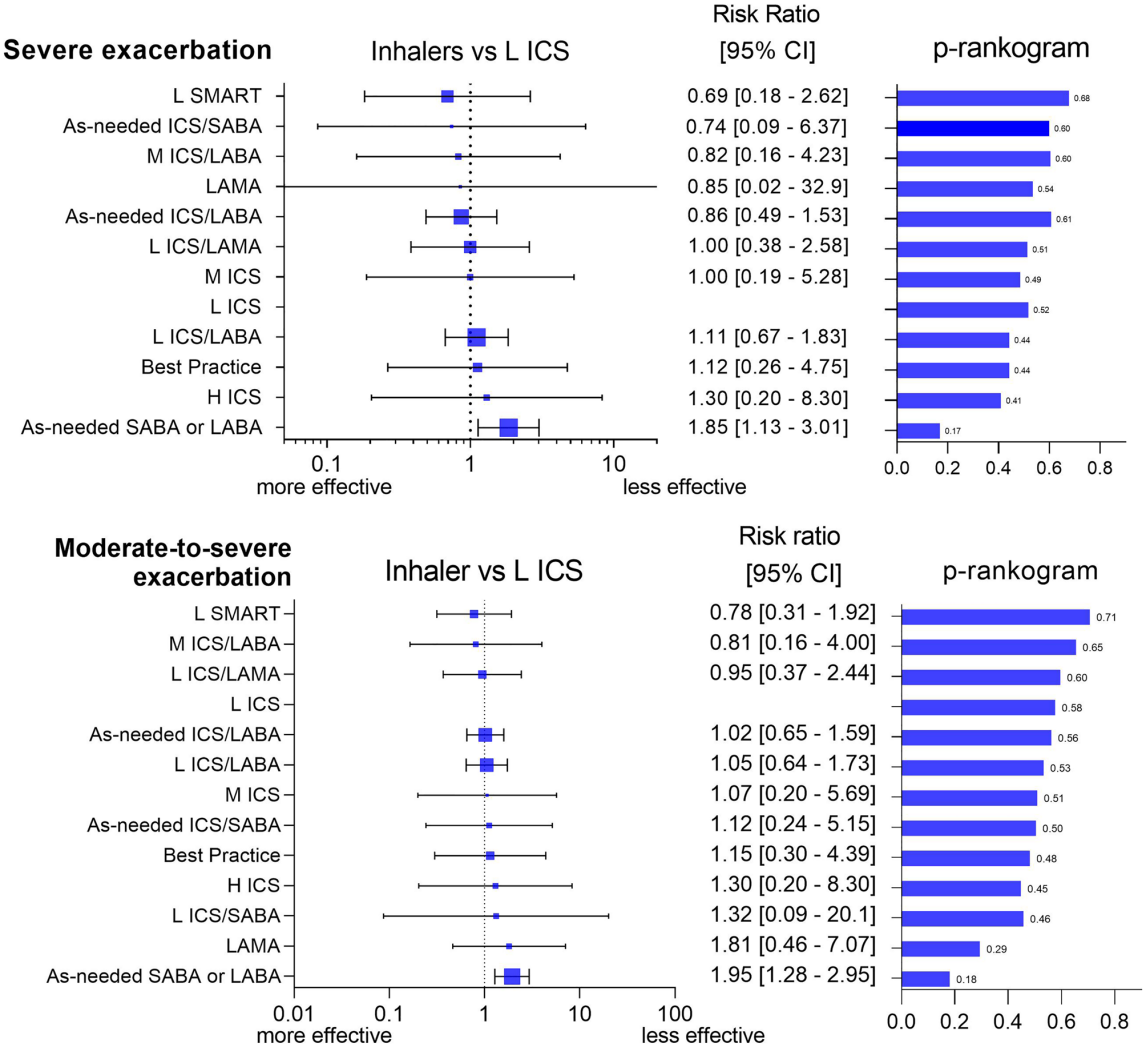
Forest plot and p-rankogram of all therapies for severe and moderate-to-severe exacerbation. Each inhaler therapy is compared with low-dose inhaled corticosteroid as maintenance. A high score in p-rankogram indicates a more potent and reliable treatment. See Fig. 2 for abbreviation.

Figure 3 shows the forest plot of therapies according to the degree of exacerbation. Low-dose ICS was used as the reference therapy because it was the recommended treatment for mild-to-moderate asthma^1^. As-needed ICS/LABA had rate ratios of 0.86 (95% credible interval [CI], 0.49–1.53) compared with low-dose ICS in severe exacerbations and 1.02 (95% CI, 0.65–1.59) in moderate-to-severe exacerbations. As-needed ICS/SABA had rate ratios of 0.74 (95% CI, 0.09–6.37) and 1.12 (95% CI, 0.24–5.15) in severe and moderate-to-severe exacerbations, respectively. The L SMART therapy had the highest rank for the prevention of both severe and moderate-to-severe exacerbations. The rate ratios of as-needed SABA or LABA were 1.85 (95% CI, 1.13–3.01) for severe exacerbations and 1.95 (95% CI, 1.28–2.95) for moderate-to-severe exacerbations.

Treatment ranking based on the p-rankogram is depicted in Fig. 3. L SMART had the highest rank for the prevention of both severe and moderate-to-severe exacerbations. As-needed ICS/LABA had the second-highest rank for the prevention of severe exacerbation, but its rank was lower for the prevention of moderate-to-severe exacerbations. Medium- or high-dose ICS did not show superiority over low-dose ICS in the prevention of exacerbations regardless of the severity. The medium-dose ICS/LABA maintenance showed the second or third-highest rank in the prevention of asthma exacerbation. As-needed SABA or LABA had the lowest rank for the prevention of both severe and moderate-to-severe exacerbations.

The estimates of between-study variance (heterogeneity) for severe (τ^2^ = 0) and moderate-to-severe (τ^2^ = 0) were low, and the inconsistency between designs was insignificant when assuming the random-effects model (P > 0.99 for both severe and moderate-to-severe exacerbations). All head-to-head comparisons for severe and moderate-to-severe exacerbations are shown in Supplementary Information. As-needed ICS/LABA was significantly superior to placebo in the indirect comparison for the prevention of severe and moderate-to-severe exacerbations. No significant difference was noted between direct and indirect modes of comparison (Supplementary Figs. S1 and S2), which was also demonstrated in the net-heat graph (Supplementary Figs. S3 and S4). The funnel plots of all direct comparisons did not show bias according to strategy type and small studies. The P value for Egger’s test was 0.37 (Supplementary Fig. S5).

### Changes in FEV_1_ according to inhaler therapy

**Figure 4:**
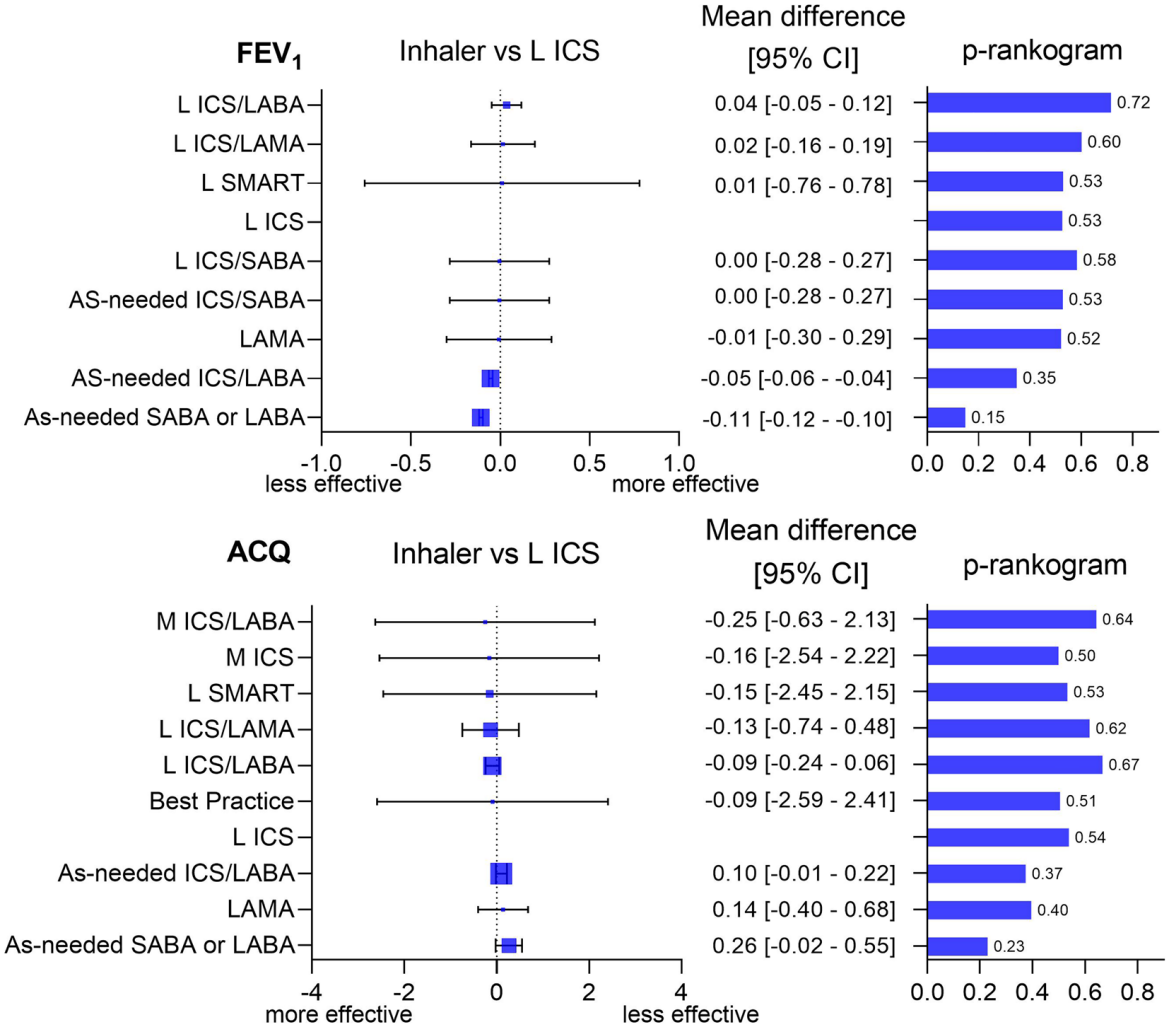
Forest plot and p-rankogram of all therapies for changes in forced expiratory volume in 1 s and Asthma Control Questionnaire. Each inhaler therapy is compared with low-dose inhaled corticosteroid as maintenance. A high score in p-rankogram indicates a more potent and reliable treatment. See Fig. 2 for abbreviation.

For changes in FEV_1_, 16 articles including 16,887 patients were included in the analysis and the network of inhaler therapies is depicted in Supplementary Fig. S6. Medium-dose ICS was not included in the analysis because it could not be compared with other inhaler therapies due to inappropriate randomization in the relevant study, as previously mentioned^32^. As-needed SABA or LABA and as-needed ICS/LABA were inferior to low-dose ICS by − 0.11 (95% CI, − 0.12 to − 0.10) and − 0.05 of mean difference (95% CI, − 0.06 to − 0.04), respectively (Fig. 4). As-needed ICS/SABA had similar efficacy with low-dose ICS, although the confidence interval was rather wider than other as-needed strategies.

Low-dose ICS/LABA had the highest rank in the p-rankogram for changes in FEV_1_ (Fig. 4). As-needed ICS/LABA and as-needed SABA or LABA were the second-lowest and lowest in the rankograms, respectively. In contrast to the ranks in exacerbation, regular ICS therapies (low-dose ICS/LABA and low-dose ICS) showed superiority to as-needed ICS/LABA in FEV_1_ change (Supplementary Information). As-needed SABA or LABA was inferior to as-needed ICS/LABA in the indirect comparison. As-needed ICS/SABA showed slight superior effect to L ICS in direct comparison; however, it showed inferiority to L ICS in indirect comparison (Supplementary Fig. S7). No significant difference between direct and indirect comparisons was observed in the net-heat graph (Supplementary Fig. S8). The comparison-adjusted funnel plot showed no significant trend in small study bias (Supplementary Fig. S9). The P value of Egger’s test was 0.40. The estimate of the between-study variance (heterogeneity) was low (τ^2^ = 0), and the inconsistency between designs was insignificant (P = 0.86).

### Changes in the ACQ score according to inhaler therapy

For the ACQ, 11 studies including 25,429 patients were included in the analysis with network graph (Supplementary Fig. S10). As-needed SABA or LABA was inferior to low-dose ICS, showing a mean difference of 0.26 (95% CI, − 0.02 to 0.55; Fig. 4). As-needed ICS/LABA was inferior to low-dose ICS by a mean difference of 0.10 (95% CI, − 0.01 to 0.22). As-needed ICS/LABA and as-needed SABA or LABA had the second-lowest and lowest rank in the p-rankogram for the changes in the ACQ score, respectively. The medium-dose ICS therapies including ICS/LABA and ICS had the highest and second-highest ranks in the forest plot, respectively, but had relatively lower ranks in the p-rankogram due to their wide confidence intervals (Fig. 4). After compositing the direct and indirect comparisons, as-needed ICS/LABA and as-needed SABA or LABA were significantly inferior to low-dose ICS/LABA but not to low-dose ICS (Supplementary Information). In the direct and indirect comparisons, the effect size was similar in comparing the therapies by net splitting (Supplementary Fig. S11). No significant difference between direct and indirect comparisons was observed in the net-heat graph (Supplementary Fig. S12). In the comparison-adjusted funnel plot, no significant bias was observed (P = 0.67; Supplementary Fig. S13). The estimates of between-study variance (heterogeneity) were low (τ^2^ = 0), and the inconsistency between designs was insignificant (P = 0.97).

### Risk of bias

Most of the included studies showed a low risk in the randomization process (92.9%), measurement of the outcome (100%), and selection of the reported results (96.4%; Supplementary Information). However, as presented in Supplementary Information, some studies showed concerns regarding deviation from the intended intervention. Most deviations of the intended intervention were due to unblinded comparison arms^33–35^ because some participants preferred non-protocol interventions^36^ that open the intervention arms to participants. Some studies controlled the participants from using non-protocol interventions by restricting the use of other inhalers or medications^37,38^. Another issue was the randomization process; Bernstein et al*.*^32^ showed similar FEV_1_ at baseline, but the mean FEV_1_ in the low-dose ICS group significantly decreased after 4 weeks of treatment, which may represent baseline bias, and no such difference was observed in other studies^4,39^; therefore, this study was excluded from the network analysis of FEV_1_ to maintain the comparability among the inhaler therapies. Despite this bias of the study, the full network of FEV_1_ could be constructed for the transparency of analysis (Supplementary Fig. S14).

## Discussion

Through this systematic review and network meta-analysis, we compared the effects of the type of inhaler therapy on exacerbation, lung function, and asthma control in mild-to-moderate asthma. Our analysis showed that as-needed ICS/LABA, which is recommended in the GINA 2019 guidelines, had a comparable effect to low-dose ICS in preventing exacerbations. However, as-needed ICS/LABA was inferior to regular ICS in terms of FEV_1_ change and ACQ score. As-needed SABA or LABA was inferior to as-needed ICS/LABA in terms of exacerbation, FEV_1_ change, and ACQ change, which supports the as-needed ICS/LABA over SABA for step 1 asthma treatment^1^.

### Comparison with other studies

Our network analysis focused on mild-to-moderate asthma because the effect of inhalers can be different according to the severity of asthma. In terms of the prevention of exacerbation, high-potency inhalers, such as medium-dose ICS or ICS/LABA, were not significantly superior to low-dose ICS-based therapies. A previous network meta-analysis reported a similar trend, in which increasing the dose of ICS had no positive effect in preventing exacerbations^14^. In contrast, a meta-analysis published in 2004 reported that higher doses of ICS were more potent in preventing exacerbations^10^; this meta-analysis suggest that a higher dose of ICS was more potent among patients with FEV_1_ of approximately 75% of the predicted value, but this result was not observed when including all asthma severities^14^. Collectively speaking, the effect of increasing the ICS dose may not always provide increased effect, especially for the prevention of exacerbation in mild asthma.

In our study, L SMART and as-needed ICS/LABA were ranked first and second, respectively, for the prevention of severe exacerbation. Until 2016, as-needed ICS/LABA was considered inferior to maintenance ICS^12^. However, after the inclusion of recent studies^4–7,32^, as-needed ICS/LABA showed a similar effect with low- and medium-dose maintenance ICS in preventing exacerbation. As-needed ICS/SABA was also tried in three studies^27,30,31^ and had similar efficacy with low-dose ICS. This suggests that as-needed ICS/SABA can be an option in the area where ICS/formoterol is not available. In a previous meta-analysis, SMART was superior to the same or a higher dose of ICS in all asthma severities^11^. A possible explanation for this finding is that asthma exacerbation can be mitigated by repeated ICS, like oral glucocorticoids in acute exacerbation^40,41^. The total dose of steroids was considered similar between L SMART and maintenance ICS^42^. Furthermore, a higher dose of regular ICS was not superior to as-needed ICS/LABA in our analysis that focused on mild-to-moderate asthma. This suggests that an increased regular ICS dose does not provide additional benefits in preventing the exacerbation of mild-to-moderate asthma and that a temporal increase depending on exacerbated symptoms is more helpful.

Previous studies showed that low-dose ICS improved FEV_1_ compared with placebo^10^ and that as-needed ICS/LABA was superior to placebo and inferior to low-dose regular ICS/LABA^15^. Our study also showed a consistent finding for FEV_1_ that as-needed ICS/LABA was superior to placebo but inferior to low-dose ICS^43^. There was no significant difference in terms of changes in FEV_1_ among the low-dose-based ICS therapies (i.e., ICS/LABA, ICS/LAMA, and ICS/SABA). Although some studies suggested that L SMART was superior to low-dose ICS or ICS/LABA^15^, such trend was not evident in our study on patients with mild-to-moderate asthma.

In terms of symptom control, previous meta-analyses did not show clear differences between inhaler therapies in mild asthma^44^ or across all severities of asthma^15^. Our study showed that low-dose ICS/LABA was superior to as-needed ICS/LABA and placebo, but the effect size was not greater than 0.5 in the ACQ, which is the minimal clinical important difference^6,7^. A similar phenomenon was observed in another meta-analysis that compared as-needed ICS/LABA and higher-dose ICS with SABA as a reliever^45^. This can be explained by the intrinsic way of how as-needed ICS/LABA is used when patients experience symptoms, thus resulting in a reduction in the ACQ score. Another possible explanation is the characteristics of patients with mild symptoms, which make it difficult to show significant differences as interventions.

### Strengths

Our study has several strengths. First, our network meta-analysis was conducted in patients with mild-to-moderate asthma, which is crucial for the assumption of transitivity. Even though inhalers with strong potency may be better for controlling severe asthma, it cannot be applicable to all severities of asthma; as such, treatments should be compared among patients with similar severity. A previous network meta-analysis included 20 studies based on the direct comparisons of as-needed ICS/LABA and SMART therapy^15^, which led to the omission of essential trials about other inhalers. To perform a more comprehensive study while following transitivity, we analyzed a higher number of studies (n = 28) by including those with indirect comparisons of as-needed therapy and limited the asthma severity to mild-to-moderate. Second, this study evaluated various outcomes including exacerbation, lung function, and symptoms and provided insight on the pros and cons of each inhaler. For example, we showed that as-needed ICS/LABA had a higher rank in exacerbation but a relatively lower rank in lung function and symptoms.

### Limitations

Our study also has some limitations. First, as the selection of studies published before 2014 was based on previous meta-analyses^12,14,15^, the comprehensiveness of our study may have been biased due to these studies. To overcome a possible inclusion bias, we referred to other meta-analyses^8,11,15,44^ and performed a manual search to find relevant studies; as a result, we were able to include 29 relevant RCTs published between 1999 and 2022. Therefore, the possibility of exclusion of essential studies from our analysis may be negligible. Second, the safety profile is unavailable in this analysis due to limited data, which warrants a further comprehensive review of the potential side effects of higher-dose inhaler therapies. Third, as we only included mild-to-moderate asthma defined by the baseline ICS dose and lung function, a higher dose of ICS or combination of inhaler therapies could not be evaluated because those treatments were not applied in clinical trials.

### Implications

As-needed ICS/LABA or ICS/SABA showed a similar effect on the prevention of exacerbation with other low-dose ICS maintenance therapies (i.e., low-dose ICS, ICS/LABA, and low-dose SMART). The mechanism of the prevention of exacerbation by as-needed ICS would be similar to that of SMART—when patients experience exacerbated symptoms, they may temporarily increase the ICS use, thereby preventing exacerbation^46^. For the recently introduced as-needed ICS/LABA, a similar efficacy in exacerbation prevention was observed despite the smaller dose of total ICS^5^. Therefore, a proper strategy of ICS usage in mild asthma may sufficiently reduce exacerbation even with lower doses of total ICS.

Meanwhile, for lung function or asthma control, the use of as-needed ICS/LABA seems less effective than regular ICS. For the restoration of lung function and control of symptoms, a higher dose of maintenance ICS may be more appropriate even in mild-to-moderate asthma. Therefore, as-needed ICS/LABA therapy may be preferred in patients with well-controlled asthma and near-normal lung function. Meanwhile, considering that as-needed ICS/LABA or ICS/SABA had a higher rank than as-needed SABA or LABA, our results support the idea that as-needed SABA should be avoided even in patients with mild asthma, as stated in the GINA guidelines^1^.

## Conclusion

As-needed ICS with LABA or SABA and as-needed ICS/LABA with maintenance ICS/LABA were effective for preventing severe exacerbation in mild-to-moderate asthma compared with the same dose of regular maintenance controllers. Meanwhile, as-needed ICS/LABA showed some weakness in the restoration of lung function and control of asthma symptoms. This difference in efficacy can serve as an important basis for tailoring the choice of inhalers according to the patients’ characteristics.

## Supplementary Information

Supplementary Information.

## Data Availability

This is a systematic review and network meta-analysis. You can access the data summarization of each study in our references.
