# Supplementary material for: Comparative efficacy of inhalers in mild-to-moderate asthma: systematic review and network meta-analysis
Source: Sci Rep. 2022 Apr 8;12:5949. doi: 10.1038/s41598-022-09941-z (PMC8993836; doi:10.1038/s41598-022-09941-z)
Supplement: Supplementary file 1 — Supplementary Information. [file 41598_2022_9941_MOESM1_ESM.pdf]

Online Supplement

Comparative efficacy of inhalers in mild-to-moderate asthma: a systematic review and network meta-analysis

Hyung Jun Park[1], Jin-Young Huh, Ji Sung Lee[2], Jae Seung Lee[1], Yeon-Mok Oh[1], and Sei Won Lee[1]

[1]Department of Pulmonary and Critical Care Medicine, Asan Medical Center, University of Ulsan College of Medicine, Seoul, South Korea

[2]Clinical Research Center, Department of Clinical Epidemiology and Biostatistics, Asan Medical Center, Seoul, South Korea

Correspondence to: Sei Won Lee, MD, PhD

Department of Pulmonary and Critical Care Medicine, Asan Medical Center, University of Ulsan College of Medicine, 88, Olympic-ro 43-gil, Songpa-gu, Seoul, 05505, South Korea

E-mail: seiwon@amc.seoul.kr

Phone: +82-2-3010-3990

Fax: +82-2-3010-6962

Tables of Contents

METHODS and INFORMATION RETRIEVAL 4

Inclusion criteria 4

Exacerbations 4

Search Strategies 5

1. PubMed 5

2. Embase 6

3. Cochrane 7

Tables 9

Supplementary Table S1. Inhalers and their doses as controller in the included studies 9

Supplementary Table S2. Baseline characteristics of the patients in the included studies 10

Supplementary Table S3. Head-to-head comparisons for severe exacerbations 12

Supplementary Table S3. Head-to-head comparisons for moderate-to-severe exacerbations 13

Supplementary Table S5. Head-to-head comparisons for FEV_1 changes of all therapies 14

Supplementary Table S6. Head-to-head comparisons for ACQ changes of all therapies 15

Supplementary Table S7. Summary of the risk of bias of the included studies 16

Supplementary Table S8. Detailed issues of the risk of bias in each included study 17

Figures 19

Supplementary Figure S1. Comparison of direct and indirect estimates for severe exacerbation. 19

Supplementary Figure S2. Comparison of direct and indirect estimates for moderate-to-severe exacerbation. 21

Supplementary Figure S3. Net heat graph of inhalers for severe exacerbation. 23

Supplementary Figure S4. Net heat graph of inhalers for moderate-to-severe exacerbation. 24

Supplementary Figure S5. Comparison-adjusted funnel plot of studies for moderate to severe exacerbations. 25

Supplementary Figure S6. Network of the strategies for FEV_1. 26

Supplementary Figure S7. Comparison of direct and indirect estimates for FEV_1 change. 27

Supplementary Figure S8. Net heat graph of inhalers for FEV_1 change. 28

Supplementary Figure S9. Comparison-adjusted funnel plot of studies for FEV_1 change. 29

Supplementary Figure S10. Network of the strategies for the ACQ. 30

Supplementary Figure S11. Comparison of direct and indirect estimates for the ACQ. 31

Supplementary Figure S12. Net heat graph of inhalers for the ACQ. 32

Supplementary Figure S13. Comparison-adjusted funnel plot of the studies for the ACQ. 33

Supplementary Figure S14. Forest plot and network of strategies with respect to change of FEV_1 (full comparison). 34

Reference 35

METHODS and INFORMATION RETRIEVAL

Inclusion criteria

The severity of asthma was defined as the difficulty in controlling asthma.[1] As no objective criteria for defining moderate or severe asthma are available, we used the lung function and Asthma Control Questionnaire (ACQ) as an indirect marker for severity. More than half of the included studies described their participants' severity. We attempted not to miss studies that included patients with mild-to-moderate asthma but did not describe their inclusion. Of the studies that described the disease severity, most had a mean forced expiratory volume in 1 s (FEV_1) of above 75% of the predicted value and mean ACQ score below 1.5. Therefore, we assumed that the aforementioned criteria for FEV_1 and the ACQ could identify mild-to-moderate asthma when the participant used below moderate doses of inhaled corticosteroid (ICS) before randomization.

Exacerbations

For defining the severity of exacerbation, several studies followed the definition of the American Thoracic Society/European Respiratory Society for severe exacerbations.[1] However, the definition of mild or moderate exacerbations was not consistent among the studies. Therefore, we defined moderate exacerbation according to the criteria used in most studies, which included deterioration in symptoms, lung function, and the increased use of rescue bronchodilator. For transitivity, we did not include mild exacerbation as much as possible to avoid bias in inhaler strategies arising from different reporting criteria.

Search Strategies

Searches in bibliographic databases

1. PubMed

   In-process, January 10, 2022

1	asthma[MeSH Terms] OR wheeze* OR bronchial spasm OR bronchial hyperreactivity OR bronchoconstric* OR respiratory hypersensitivity
2	randomized controlled trials[MeSH Major Topic] OR randomized controlled trial OR random allocation OR double blind method OR single blind method OR clinical trial
3	case report OR letter OR historical article OR review, multicase OR review of reported cases
4	Budesonide, Formoterol Fumarate Drug Combination[MeSH Terms] OR symbicort OR Budesonide* OR Beclometasone* OR Mometasone Furoate, Formoterol Fumarate Drug Combination[MeSH Terms] OR dulera OR zenhale OR fluticasone* OR ciclesonide* OR betamethasone* OR mometasone*
5	bitolterol OR carbuterol OR fenoterol OR isoetharine OR bronkometer OR pirbuterol OR maxair OR reproterol OR rimiterol OR salbutamol OR Albuterol[MeSH Terms] OR ventolin OR levosalbutamol OR terbutaline OR brethine OR bricanyl OR brethaire OR tulobuterol OR metaproterenol OR SABA))) OR ((formoterol OR Formoterol Fumarate[MeSH Terms] OR eformoterol OR atimos OR foradil OR oxis OR perforomist OR LABA
6	1 AND 2 NOT 3

7	4 OR 5
8	6 AND 7

2. Embase

In-process, January 10, 2022

1	“asthma”/exp OR “wheezing”/exp OR “bronchospasm”/exp OR “bronchoconstriction”/exp OR “bronchus hyperreactivity”/exp OR “bronchial hyperresponsiveness”/exp
2	(“randomized controlled trial”/exp OR “randomized controlled trial” OR “randomized controlled trial (topic)”/exp OR “randomized controlled trial (topic)” OR “randomization”/exp OR randomization OR “double blind procedure”/exp OR “double blind procedure” OR “clinical trial”/exp OR “clinical trial” OR “placebo”/exp OR placebo)
3	(case AND report) OR letter OR (historical AND article) OR (review AND of AND reported AND case) OR review
4	#1 AND #2 NOT #3
5	inhaled AND corticosteroid OR “beclometasone dipropionate” OR fluticasone* OR budesonide* OR betamethasone* OR flunisolide* OR mometasone* OR ciclesonide*
6	“bitolterol”/exp OR bitolterol OR carbuterol:jt OR fenoterol:au OR isoetharine:af OR “bronkosol”/exp OR bronkosol OR “bronkometer”/exp OR bronkometer OR

	“pirbuterol”/exp OR pirbuterol OR “maxair”/exp OR maxair OR “reproterol”/exp OR reproterol OR “rimiterol”/exp OR rimiterol OR “salbutamol”/exp OR salbutamol OR “albuterol”/exp OR albuterol OR “ventolin”/exp OR ventolin OR “levalbuterol”/exp OR levalbuterol OR “brethine”/exp OR brethine OR “terbutaline”/exp OR terbutaline OR “hokunalin”/exp OR hokunalin OR “saba”/exp OR saba
7	formoterol OR atimos OR foradil OR oxis OR “perforomist”/exp OR perforomist OR laba
8	#5 OR #6 OR #7
9	#4 AND #8

3. Cochrane

1	MeSH descriptor: [Asthma] explode all trees OR wheeze* OR bronchial spasm OR asthma OR bronchoconstric* OR respiratory hypersensitivity
2	MeSH descriptor: [Randomized Controlled Trial] explode all trees OR double blind OR single blind OR random allocation OR randomized controlled trial OR clinical trial
3	case report OR letter OR historical article OR MeSH descriptor: [Review] this term only OR review of reported cases
4	#1 OR #2 NOT #3
5	Budesonide OR symbicort OR beclomethasone OR MeSH descriptor: [Budesonide] explode all trees OR MeSH descriptor: [Budesonide, Formoterol Fumarate Drug

	Combination] explode all trees OR beclometasone OR mometasone OR MeSH descriptor: [Mometasone Furoate] explode all trees OR MeSH descriptor: [Mometasone Furoate, Formoterol Fumarate Drug Combination] explode all trees OR dulera OR zenhale OR fluticasone OR ciclesonide OR betametasone
6	Bitolterol OR carbuterol OR fenoterol OR isoetharine OR bronkometer OR pirbuterol OR maxair OR reproterol OR rimiterol OR salbutamol OR albuterol OR ventolin OR levosalbutamol OR terbutaline OR brethine OR bricanyl OR brethaire OR tulobuterol OR metaproterenol OR SABA
7	Eformoterol OR atimos OR foradil OR oxis OR perforomist OR LABA
#8	#5 OR #6 OR #7
9	#4 AND #8

Tables

Supplementary Table S1. Inhalers and their doses as controller in the included studies

Class	Type of controller	Daily dose (μg)
As-needed ICS/LABA	Budesonide/formoterol	160/4.5
As-needed ICS/SABA	Beclomethasone/albuterol	250/100
	Beclomethasone/albuterol	40-80/180(for children)
L ICS/LABA	Fluticasone propionate/salmeterol	100/50 - 200/100
	Fluticasone furoate/vilanterol	100/25
	Budesonide/formoterol*	100/4.5 - 400/9
	Mometasone/formoterol*	100-200
L ICS	Budesonide	200–400
	Fluticasone propionate	100–250
	Fluticasone furoate	100
L ICS/SABA	Beclometasone/albuterol	500/200
L ICS/LAMA	Fluticasone/tiotropium*	100–250/18
	Budesonide/tiotropium*	80–160/18
	Mometasone/tiotropium*	100–200/18
L SMART	Budesonide/formoterol	80/4.5 - 400/12 + 160/4.5
	Beclomethasone/albuterol	80 – 160
M ICS	Fluticasone propionate	500
	Budesonide	640
M ICS/LABA	Budesonide/formoterol	640–800/18–24
H ICS	Fluticasone propionate	880
Best Practice	Adjustable dose of inhaler based on symptoms	
As-needed SABA or LABA (placebo)	Albuterol/salbutamol	100-200
	Terbutaline	500
	Formoterol	4.5

L, low-dose; ICS, inhaled corticosteroid; LABA, long-acting β2 agonist; LAMA, long-acting muscarinic antagonist; M, medium-dose; H, high-dose.
*Wechsler et al.[2]: ICS/LABA was compared with various ICS/LABA and ICS/LAMA. The included ICS types were fluticasone (100–500), budesonide (80–160), and mometasone (100–200). As most (90%) of the ICS was low-dose ICS, this ICS/LABA group was considered as low-dose ICS/LABA, as the same criteria applied to ICS/LAMA and ICS/LAMA.

Supplementary Table S2. Baseline characteristics of the patients in the included studies

Study ID	Patients (n)	Mean age (year)	Follow-up (week)	Severity of asthma*	FEV_1 of predicted (%)	ACQ-5	Treatment 1	Treatment 2	Treatment 3	Treatment 4
Bailey 2008[3]	475	31	52	N/A	77	N/A	L ICS/LABA	L ICS		
Beasley 2015[4]	4215	40.7	52	mild	84.3	1.51	As-needed ICS/LABA	L ICS		
Bateman 2018[5]	1519	42	52	N/A	75.3	1.7	M ICS/LABA	M ICS		
Beasley 2019[6]	668	35	52	mild	89	1.1	As-needed ICS/LABA	L ICS	Placebo	
Bernstein 2018[7]	1504	43.5	24	N/A	90.2	N/A	L ICS/LABA	M ICS/LABA	M ICS	
Busse 2014[8]	347	36.4	24	Mild/moderate	81.02	N/A	L ICS (FF 50)	L ICS	Placebo	
Chuchalin 2008[9]	2258	35	52	Mild	98	N/A	L ICS/LABA	L ICS	Placebo	
FitzGerald 2005[10]	688	45	52	N/A	82	N/A	M ICS/LABA	Best Practice		
Haahtela 2006[11]	92	37	24	Intermittent	101	1.52	As-needed ICS/LABA	Placebo		
Hardy, Jo 2019[12]	885	42	52	Mild	87	1.1	As-needed ICS/LABA	L ICS		
Jackson 2018[13]	254	8	48	Mild/moderate	90	N/A	L ICS	H ICS		
Lundborg 2006[14]	491	40	26	Moderate	95	0.93	L SMART	M ICS/LABA		
Nathan 1999[15]	288	11	44	Mild	101	N/A	L ICS	Placebo		
Martinez 2011[16]	386	30	26	N/A	78	N/A	L SMART	As-needed ICS/SABA	L ICS	Placebo
O'Bryrne 2018[17]	3849	40	52	Mild	84	1.5	As-needed ICS/LABA	L ICS	Placebo	
Papi 2015[18]	866	43	52	Moderate	94	0.55	As-needed ICS/LABA	L ICS/LABA		
Papi 2007[19]	466	39	26	N/A	88	N/A	As-needed ICS/SABA	L ICS	Placebo	L ICS/SABA
Pauwels 2003[20]	7241	24	154	Mild	86	N/A	L ICS	Placebo		
Peter 2016[21]	11693	43	26	Persistent	N/A	1.2	L ICS	L ICS/LABA	M ICS/LABA	M ICS
Postma 2011[22]	657	30	52	Mild	95	N/A	L ICS	L ICS/LABA	placebo	
Rabe 2006[23]	697	38	24	Mild/moderate	75	N/A	L SMART	L ICS		
Reddel 2008[24]	44	40	47	Mild	98	N/A	L ICS	Placebo		
Renzi 2010[25]	526	34	24	Mild	92.8	N/A	L ICS	L ICS/LABA		

Riemersma 2012[26]	102	44	52	Mild/moderate	96	0.8	L SMART	Best Practice		
Sovani 2008[27]	71	40	24	N/A	85	1.95	L ICS	L SMART		
Stallberg 2008[28]	1776	43	52	N/A	95	N/A	L SMART	L ICS/LABA	Best Practice	
Sumino 2020[29]	206	10.2	52	Mild	99.2	N/A	As-needed ICS/SABA	L ICS		
Wechsler 2015[2]	1070	45	77	N/A	78	2.11	L ICS/LAMA	L ICS/LABA		
Cindy 2020[30]	181	9.1	24	Mild	75.1	N/A	L ICS/LABA	L ICS		

ACQ-5, asthma control questionnaire; N/A, not accessible. See Supplementary Table S1 for other abbreviations.

*Described as the studies presented.

Supplementary Table S3. Head-to-head comparisons for severe exacerbations

As-needed (ICS/LABA)	.	0.45 (0.16 to 1.25)	.	.	0.87 (0.48 to 1.57)	.	.	.	.	.	.
1.17 (0.13 to 10.9)	As-needed (ICS/SABA)	.	.	.	0.74 (0.09 to 6.37)	.	.	.	.	.	.
0.47 (0.24 to 0.92)	0.40 (0.04 to 3.64)	As-needed SABA or LABA	.	.	1.89 (1.13 to 3.16)	1.54 (0.66 to 3.60)	.	.	2.18 (0.06 to 81.81)	.	.
0.77 (0.16 to 3.62)	0.66 (0.05 to 8.81)	1.65 (0.37 to 7.34)	Best Practice	.	.	0.95 (0.18 to 5.01)	.	1.36 (0.31 to 5.98)	.	.	1.60 (0.34 to 7.55)
0.66 (0.10 to 4.62)	0.57 (0.03 to 9.75)	1.42 (0.21 to 9.65)	0.86 (0.08 to 9.03)	H ICS	1.30 (0.20 to 8.30)	.	.	.	.	.	.
0.86 (0.49 to 1.53)	0.74 (0.09 to 6.37)	1.85 (1.13 to 3.01)	1.12 (0.26 to 4.75)	1.30 (0.20 to 8.30)	L ICS	0.88 (0.52 to 1.49)	.	3.11 (0.20 to 47.89)	.	.	.
0.78 (0.37 to 1.65)	0.67 (0.07 to 6.10)	1.67 (0.89 to 3.14)	1.01 (0.26 to 3.99)	1.18 (0.17 to 8.03)	0.90 (0.55 to 1.50)	L ICS/LABA	1.11 (0.50 to 2.49)	1.37 (0.30 to 6.20)	.	0.79 (0.07 to 9.04)	0.99 (0.08 to 13.15)
0.87 (0.29 to 2.60)	0.74 (0.07 to 7.82)	1.85 (0.67 to 5.15)	1.13 (0.23 to 5.52)	1.31 (0.16 to 10.49)	1.00 (0.39 to 2.60)	1.11 (0.50 to 2.49)	L ICS/LAMA	.	.	.	.
1.25 (0.30 to 5.30)	1.07 (0.09 to 13.5)	2.67 (0.66 to 10.8)	1.62 (0.41 to 6.46)	1.88 (0.19 to 18.45)	1.45 (0.38 to 5.49)	1.60 (0.45 to 5.74)	1.44 (0.32 to 6.52)	L SMART	.	.	.
1.02 (0.03 to40.80)	0.87 (0.01 to 60.86)	2.18 (0.06 to 81.81)	1.32 (0.03 to 66.79)	1.53 (0.03 to 92.7)	1.18 (0.03 to 45.80)	1.30 (0.03 to 51.77)	1.17 (0.03 to 50.83)	0.81 (0.02 to 39.63)	LAMA	.	.
0.87 (0.15 to 5.01)	0.74 (0.05 to 11.28)	1.85 (0.33 to 10.23)	1.12 (0.27 to 4.60)	1.30 (0.11 to 15.8)	1.00 (0.19 to 5.30)	1.11 (0.22 to 5.48)	1.00 (0.17 to 5.98)	0.69 (0.12 to 4.08)	0.85 (0.02 to 46.89)	M ICS	1.20 (0.74 to 1.95)
1.05 (0.19 to 5.89)	0.90 (0.06 to 13.39)	2.24 (0.42 to 12.01)	1.36 (0.35 to 5.24)	1.58 (0.13 to 18.7)	1.21 (0.24 to 6.22)	1.34 (0.28 to 6.43)	1.21 (0.21 to 7.03)	0.84 (0.15 to 4.75)	1.03 (0.02 to56.01)	1.21 (0.75 to 1.96)	M ICS/LABA

Interventions are reported in alphabetical order. The upper triangle displays the pooled effect size of the direct comparisons available in our network. The lower triangle contains the network analysis effect size for each comparison. Data in columns are relative risk (95% CI) of each comparison. *The mark means statistical significant ($p < 0.05$). Placebo, as-needed SABA or LABA.

Supplementary Table S3. Head-to-head comparisons for moderate-to-severe exacerbations

As-needed (ICS/LABA)	.	.	.	0.90<br>(0.54 to 1.50)	1.49<br>(0.63 to 3.55)	.	.	.	.	.	.	0.47<br>(0.23 to 0.94)
0.91<br>(0.19 to 4.38)	As-needed (ICS/SABA)	.	.	1.29<br>(0.18 to 9.18)	.	.	0.51<br>(0.01 to 26.7)	1.19<br>(0.15 to 9.51)	.	.	.	0.63<br>(0.12 to 3.38)
0.89<br>(0.22 to 3.52)	0.97<br>(0.14 to 6.96)	Best Practice	.	.	0.95<br>(0.18 to 5.01)	.	.	1.36<br>(0.31 to 5.98)	.	.	1.60<br>(0.34 to 7.55)	.
0.78<br>(0.12 to 5.26)	0.86<br>(0.08 to 9.50)	0.88<br>(0.09 to 8.70)	H ICS	1.30<br>(0.20 to 8.30)	.	.	.	.	.	.	.	.
1.02<br>(0.65 to 1.59)	1.12<br>(0.24 to 5.15)	1.15<br>(0.30 to 4.39)	1.30<br>(0.20 to 8.30)	L ICS	0.77<br>(0.41 to 1.44)	.	0.56<br>(0.01 to 29.7)	1.41<br>(0.43 to 4.63)	.	.	.	0.50<br>(0.32 to 0.78)
0.97<br>(0.55 to 1.69)	1.06<br>(0.22 to 5.13)	1.09<br>(0.30 to 4.02)	1.24<br>(0.18 to 8.42)	0.95<br>(0.58 to 1.56)	L ICS/LABA	1.11<br>(0.50 to 2.49)	.	1.37<br>(0.30 to 6.20)	.	0.79<br>(0.07 to 9.04)	0.99<br>(0.08 to 13.2)	0.65<br>(0.28 to 1.52)
1.07<br>(0.40 to 2.86)	1.18<br>(0.20 to 6.92)	1.21<br>(0.26 to 5.61)	1.37<br>(0.17 to 11.0)	1.06<br>(0.41 to 2.72)	1.11<br>(0.50 to 2.49)	L ICS/LAMA	.	.	.	.	.	.
0.77<br>(0.05 to 11.9)	0.84<br>(0.04 to 16.4)	0.87<br>(0.04 to 17.7)	0.98<br>(0.04 to 26.5)	0.76<br>(0.05 to 11.5)	0.80<br>(0.05 to 12.5)	0.72<br>(0.04 to 12.6)	L ICS/SABA	.	.	.	.	0.55<br>(0.03 to 9.57)
1.31<br>(0.49 to 3.46)	1.44<br>(0.28 to 7.43)	1.48<br>(0.40 to 5.46)	1.67<br>(0.21 to 13.2)	1.29<br>(0.52 to 3.18)	1.35<br>(0.53 to 3.43)	1.22<br>(0.36 to 4.17)	1.70<br>(0.10 to 29.2)	L SMART	.	.	.	0.66<br>(0.09 to 4.84)
0.56<br>(0.14 to 2.26)	0.62<br>(0.08 to 4.55)	0.63<br>(0.10 to 4.18)	0.72<br>(0.07 to 7.16)	0.55<br>(0.14 to 2.15)	0.58<br>(0.14 to 2.39)	0.52<br>(0.10 to 2.66)	0.73<br>(0.04 to 14.7)	0.43<br>(0.09 to 2.14)	LAMA	.	.	0.93<br>(0.25 to 3.41)
0.95<br>(0.17 to 5.22)	1.05<br>(0.11 to 9.67)	1.08<br>(0.24 to 4.76)	1.22<br>(0.10 to 14.9)	0.94<br>(0.18 to 5.01)	0.99<br>(0.19 to 5.03)	0.89<br>(0.14 to 5.47)	1.24<br>(0.05 to 29.9)	0.73<br>(0.13 to 4.10)	1.70<br>(0.20 to 14.4)	M ICS	1.29<br>(0.61 to 2.76)	.
1.25<br>(0.25 to 6.35)	1.38<br>(0.16 to 12.0)	1.41<br>(0.37 to 5.42)	1.60<br>(0.14 to 18.5)	1.23<br>(0.25 to 6.09)	1.30<br>(0.28 to 6.10)	1.17<br>(0.20 to 6.69)	1.63<br>(0.07 to 37.7)	0.96<br>(0.19 to 4.94)	2.24<br>(0.28 to 17.8)	1.31<br>(0.62 to 2.79)	M ICS/LABA	.
0.52<br>(0.31 to 0.87)	0.57<br>(0.13 to 2.63)	0.59<br>(0.15 to 2.32)	0.67<br>(0.10 to 4.47)	0.51<br>(0.34 to 0.78)	0.54<br>(0.31 to 0.95)	0.49<br>(0.18 to 1.30)	0.68<br>(0.05 to 10.2)	0.40<br>(0.15 to 1.03)	0.93<br>(0.25 to 3.41)	0.55<br>(0.10 to 2.99)	0.42<br>(0.08 to 2.10)	Placebo

Interventions are reported in alphabetical order. The upper triangle displays the pooled effect size of the direct comparisons available in our network. The lower triangle contains the network analysis effect size for each comparison. Data in columns are relative risk (95% CI) of each comparison. *The mark means statistical significant ($p < 0.05$). Placebo, as-needed SABA or LABA.

Supplementary Table S5. Head-to-head comparisons for FEV_1 changes of all therapies

AS-needed (ICS/LABA)		-0.05* (-0.06 to -0.04)	-0.15 (-0.37 to 0.08)					0.05 (0.04 to 0.06)
-0.05 (-0.33 to 0.23)	AS-needed (ICS/SABA)	0.03 (-0.29 to 0.35)			0.00 (-0.32 to 0.32)			0.07 (-0.25 to 0.39)
-0.05* (-0.06 to -0.04)	-0.00 (-0.28 to 0.27)	L ICS	-0.03 (-0.12 to 0.06)		-0.03 (-0.35 to 0.29)	-0.01 (-0.78 to 0.76)		0.11 (0.10 to 0.12)
-0.09* (-0.17 to -0.01)	-0.04 (-0.33 to 0.25)	-0.04 (-0.12 to 0.05)	L ICS/LABA	0.02 (-0.14 to 0.18)				0.20 (-0.04 to 0.44)
-0.07 (-0.25 to 0.11)	-0.02 (-0.35 to 0.31)	-0.02 (-0.19 to 0.16)	0.02 (-0.14 to 0.18)	L ICS/LAMA				
-0.05 (-0.33 to 0.23)	-0.00 (-0.32 to 0.32)	0.00 (-0.27 to 0.28)	0.04 (-0.25 to 0.33)	0.02 (-0.31 to 0.35)	L ICS/SABA			0.07 (-0.25 to 0.39)
-0.06 (-0.83 to 0.70)	-0.01 (-0.83 to 0.80)	-0.01 (-0.78 to 0.76)	0.03 (-0.75 to 0.80)	0.01 (-0.78 to 0.79)	-0.01 (-0.83 to 0.80)	L SMART		
-0.05 (-0.34 to 0.25)	0.00 (-0.40 to 0.41)	0.01 (-0.29 to 0.30)	0.04 (-0.26 to 0.35)	0.02 (-0.32 to 0.37)	0.00 (-0.40 to 0.41)	0.02 (-0.81 to 0.84)	LAMA	0.10 (-0.19 to 0.39)
0.05* (0.04 to 0.06)	0.10 (-0.17 to 0.38)	0.11* (0.10 to 0.12)	0.14* (0.06 to 0.23)	0.12 (-0.05 to 0.30)	0.10 (-0.17 to 0.38)	0.12 (-0.65 to 0.89)	0.10 (-0.19 to 0.39)	Placebo

Interventions are reported in alphabetical order. The upper triangle displays the pooled effect size of the direct comparisons available in our network. The lower triangle contains the network analysis effect size for each comparison. Data in columns are the mean difference of FEV_1 (95% CI) of each comparison. *The mark means statistical significant ($p < 0.05$). Placebo: as-needed SABA or LABA.

Supplementary Table S6. Head-to-head comparisons for ACQ changes of all therapies

As-needed (ICS/LABA)		0.10 (-0.02 to 0.22)	0.21 (-0.29 to 0.71)						-0.14 (-0.46 to 0.18)
0.19 (-2.31 to 2.69)	Best Practice				0.06 (-0.90 to 1.02)				
0.10 (-0.01 to 0.22)	-0.09 (-2.59 to 2.41)	L ICS	0.09 (-0.06 to 0.24)		0.15 (-2.15 to 2.45)				-0.28 (-0.61 to 0.04)
0.19* (0.01 to 0.37)	0.00 (-2.50 to 2.50)	0.09 (-0.06 to 0.24)	L ICS/LABA	0.04 (-0.56 to 0.64)					
0.23 (-0.39 to 0.86)	0.04 (-2.53 to 2.61)	0.13 (-0.48 to 0.74)	0.04 (-0.56 to 0.64)	L ICS/LAMA					
0.25 (-2.06 to 2.56)	0.06 (-0.90 to 1.02)	0.15 (-2.15 to 2.45)	0.06 (-2.25 to 2.37)	0.02 (-2.37 to 2.40)	L SMART			0.10 (-0.48 to 0.68)	
-0.03 (-0.57 to 0.50)	-0.23 (-2.78 to 2.33)	-0.14 (-0.68 to 0.40)	-0.23 (-0.79 to 0.33)	-0.27 (-1.08 to 0.55)	-0.29 (-2.65 to 2.08)	LAMA			-0.13 (-0.58 to 0.33)
0.26 (-2.12 to 2.64)	0.07 (-1.05 to 1.19)	0.16 (-2.22 to 2.54)	0.07 (-2.31 to 2.45)	0.03 (-2.43 to 2.48)	0.01 (-0.57 to 0.59)	0.30 (-2.14 to 2.73)	M ICS	0.09* (0.00 to 0.18)	
0.35 (-2.03 to 2.73)	0.16 (-0.96 to 1.28)	0.25 (-2.13 to 2.63)	0.16 (-2.22 to 2.54)	0.12 (-2.34 to 2.57)	0.10 (-0.48 to 0.68)	0.39 (-2.05 to 2.82)	0.09* (0.00 to 0.18)	M ICS/LABA	
-0.16 (-0.45 to 0.12)	-0.35 (-2.87 to 2.16)	-0.26 (-0.55 to 0.02)	-0.36* (-0.68 to -0.04)	-0.40 (-1.07 to 0.28)	-0.41 (-2.74 to 1.91)	-0.13 (-0.58 to 0.33)	-0.42 (-2.82 to 1.97)	-0.51 (-2.91 to 1.88)	Placebo

Interventions are reported in alphabetical order. The upper triangle displays the pooled effect size of the direct comparisons available in our network. The lower triangle contains the network analysis effect size for each comparison. Data in columns are the mean difference of ACQ (95% CI) of each comparison. *The mark means statistical significant ($p < 0.05$). Placebo, as-needed SABA or LABA.

Supplementary Table S7. Summary of the risk of bias of the included studies

	Randomization process	Deviations from intended interventions	Missing outcome data	Measurement of the outcome	Selection of the reported result	Overall bias
Low risk	92.9	78.6	92.9	100	96.4	71.4
Some concerns	3.6	17.9	7.1	0	3.6	25
High risk	3.6	3.6	0	0	0	3.6

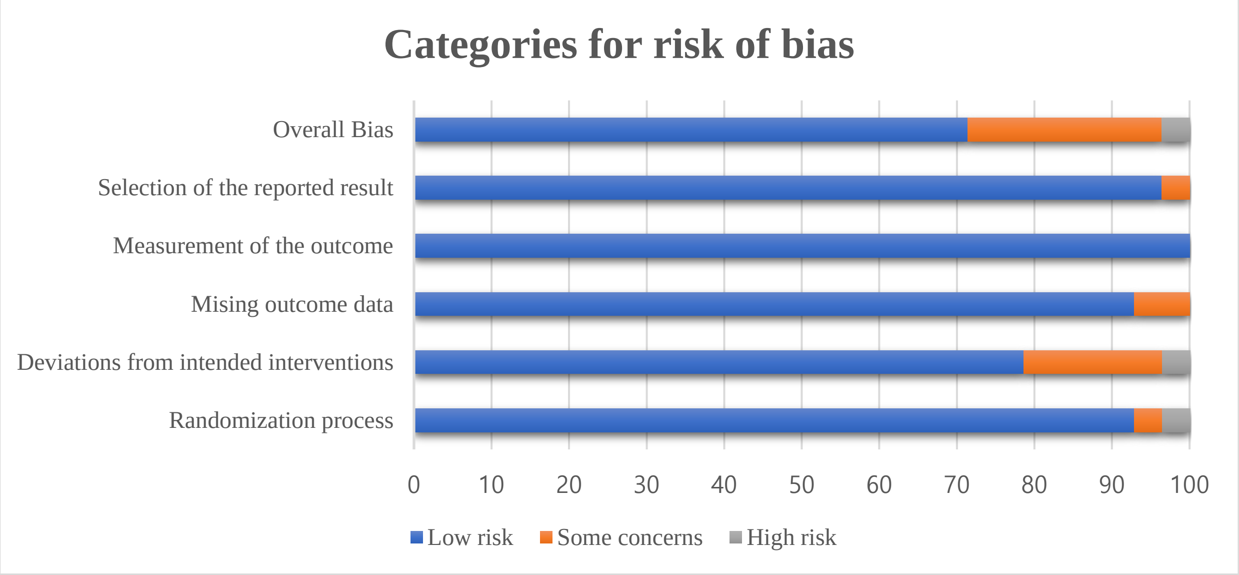

Supplementary Table S8. Detailed issues of the risk of bias in each included study

Study ID	Outcome	Randomization process	Deviations from intended interventions	Missing outcome data	Measurement of the outcome	Selection of the reported result	Overall
Bailey 2008[3]	FEV1	+	+	+	+	+	+
Beasley 2015[4]	Exacerbation, ACQ	+	+	+	+	+	+
Bateman 2018[5]	Exacerbation, FEV_1, ACQ	+	+	+	+	+	+
Beasley 2019[6]	Exacerbation, FEV_1, ACQ	+	?	+	+	+	!
Bernstein 2018[7]	Exacerbation, FEV_1	?	?	+	+	+	?
Busse 2014[8]	Exacerbation, FEV_1	+	+	+	+	+	+
Chuchalin 2008[9]	FEV_1	+	+	+	+	+	+
FitzGerald 2005[10]	Exacerbation	+	+	+	+	+	+
Haahtela 2006[11]	FEV_1	+	?	+	+	?	!
Hardy, Jo 2019[12]	Exacerbation, FEV_1, ACQ	+	+	+	+	+	+
Jackson 2018[13]	Exacerbation	+	+	+	+	+	+
Lundborg 2006[14]	ACQ	+	?	?	+	+	!
Nathan 1999[15]	FEV_1	+	+	+	+	+	+
Martinez 2011[16]	Exacerbation	+	+	+	+	+	+
O'Bryrne 2018[17]	Exacerbation, FEV_1, ACQ	+	+	+	+	+	+
Papi 2015[18]	Exacerbation, FEV_1, ACQ	+	+	?	+	+	!
Papi 2007[19]	Exacerbation, FEV_1, ACQ	+	+	+	+	+	+
Pauwels 2003[20]	Exacerbation	+	+	+	+	+	+
Peter 2016[21]	Exacerbation, ACQ	+	+	+	+	+	+
Postma 2011[22]	FEV_1	+	?	+	+	+	!

Rabe 2006[23]	Exacerbation	?	+	+	+	+	!
Reddel 2008[24]	Exacerbation, FEV_1	+	+	+	+	+	+
Renzi 2010[25]	Exacerbation, FEV_1	+	+	+	+	+	+
Riemersma 2012[26]	Exacerbation, ACQ	+	+	+	+	+	+
Sovani 2008[27]	FEV_1, ACQ	+	+	+	+	+	+
Stallberg 2008[28]	Exacerbation	+	?	+	+	+	!
Wechsler 2015[2]	Exacerbation, FEV_1, ACQ	+	+	+	+	+	+
Sumino 2020[29]	Exacerbation	+	+	+	+	+	+
Cindy 2020[30]	Exacerbation	+	+	+	+	+	+

+ , low risk; ? , some concerns; ? , high risk

Figures

Supplementary Figure S1. Comparison of direct and indirect estimates for severe exacerbation.

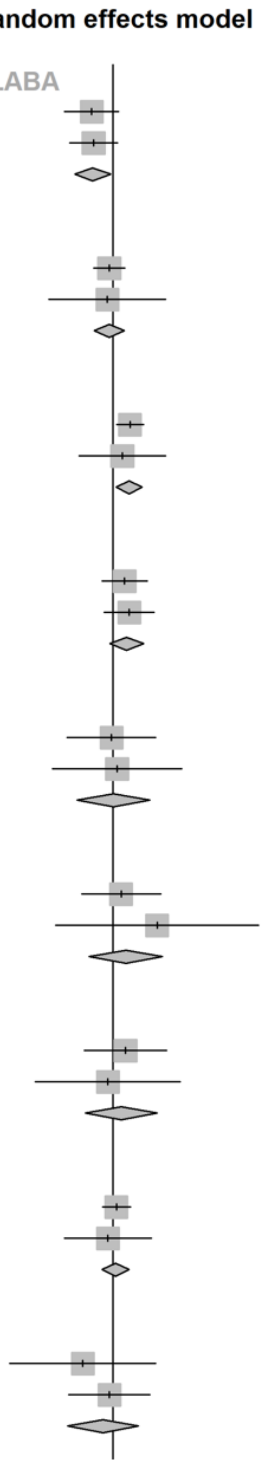

Comparison	Number of Studies	Direct Evidence	I2	RR	95%-CI
**As-needed (ICS/LABA) vs As-needed SABA or LABA**					
Direct estimate	2	0.44	0	0.45	[0.16; 1.25]
Indirect estimate				0.48	[0.20; 1.20]
Network estimate				0.47	[0.24; 0.92]
**As-needed (ICS/LABA) vs L ICS**					
Direct estimate	4	0.93	0	0.87	[0.48; 1.57]
Indirect estimate				0.81	[0.09; 7.26]
Network estimate				0.86	[0.49; 1.53]
**As-needed SABA or LABA vs L ICS**					
Direct estimate	7	0.91	0	1.89	[1.13; 3.16]
Indirect estimate				1.42	[0.28; 7.27]
Network estimate				1.85	[1.13; 3.01]
**As-needed SABA or LABA vs L ICS/LABA**					
Direct estimate	1	0.55		1.54	[0.66; 3.60]
Indirect estimate				1.84	[0.72; 4.71]
Network estimate				1.67	[0.89; 3.14]
**Best Practice vs L ICS/LABA**					
Direct estimate	1	0.68		0.95	[0.18; 5.01]
Indirect estimate				1.17	[0.10; 13.13]
Network estimate				1.01	[0.26; 3.99]
**Best Practice vs L SMART**					
Direct estimate	2	0.87	0	1.36	[0.31; 5.98]
Indirect estimate				5.22	[0.12; 233.81]
Network estimate				1.62	[0.41; 6.46]
**Best Practice vs M ICS/LABA**					
Direct estimate	1	0.75		1.60	[0.34; 7.55]
Indirect estimate				0.83	[0.05; 12.57]
Network estimate				1.36	[0.35; 5.24]
**L ICS/LABA vs L ICS**					
Direct estimate	4	0.90	0	1.14	[0.67; 1.94]
Indirect estimate				0.83	[0.16; 4.24]
Network estimate				1.11	[0.67; 1.83]
**L SMART vs L ICS**					
Direct estimate	1	0.24		0.32	[0.02; 4.95]
Indirect estimate				0.88	[0.19; 4.03]
Network estimate				0.69	[0.18; 2.62]

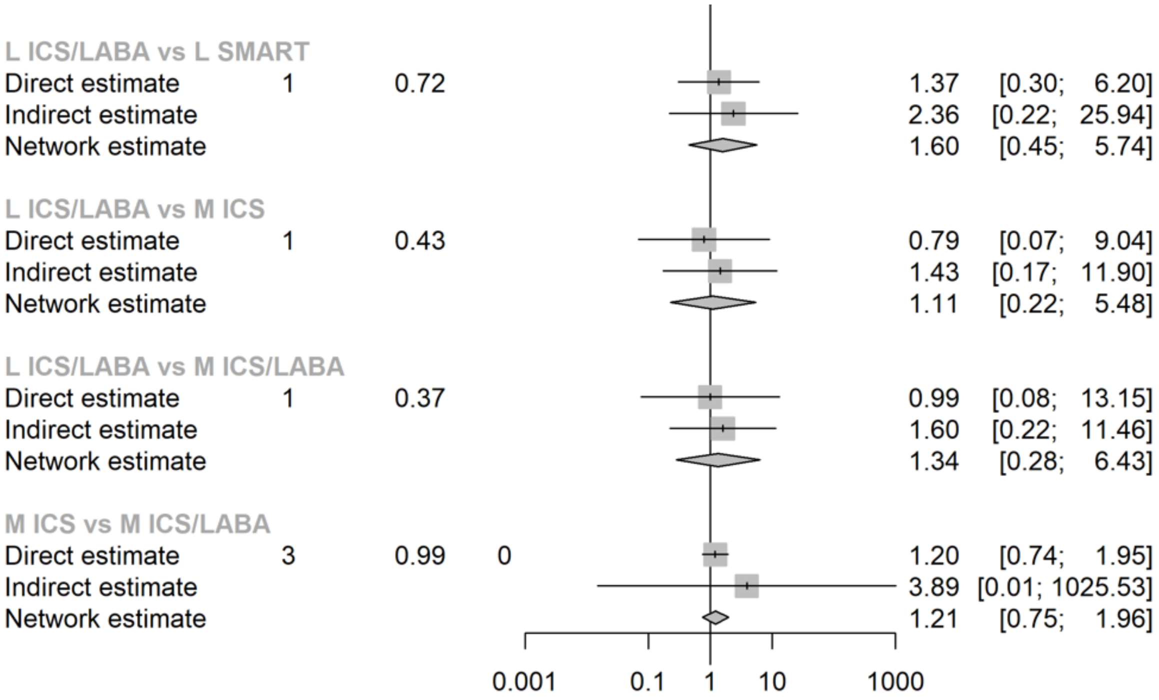

Placebo, as-needed SABA or LABA.

Supplementary Figure S2. Comparison of direct and indirect estimates for moderate-to-severe exacerbation.

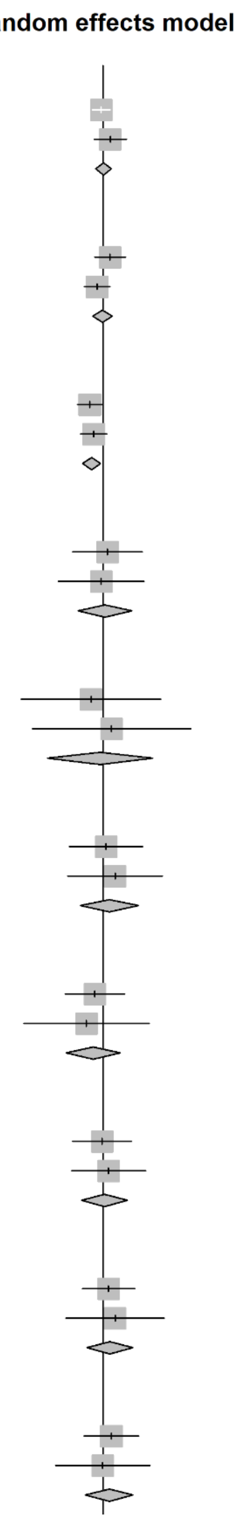

Comparison	Number of Studies	Direct Evidence	I2	RR	95%-CI
**As-needed (ICS/LABA) vs L ICS**					
Direct estimate	4	0.76	0	0.90	[0.54; 1.50]
Indirect estimate				1.50	[0.60; 3.75]
Network estimate				1.02	[0.65; 1.59]
**As-needed (ICS/LABA) vs L ICS/LABA**					
Direct estimate	1	0.41		1.49	[0.63; 3.55]
Indirect estimate				0.71	[0.35; 1.47]
Network estimate				0.97	[0.55; 1.69]
**As-needed (ICS/LABA) vs placebo**					
Direct estimate	2	0.54	0	0.47	[0.23; 0.94]
Indirect estimate				0.59	[0.28; 1.24]
Network estimate				0.52	[0.31; 0.87]
**As-needed (ICS/SABA) vs L ICS**					
Direct estimate	2	0.61	0	1.29	[0.18; 9.18]
Indirect estimate				0.90	[0.08; 10.25]
Network estimate				1.12	[0.24; 5.15]
**As-needed (ICS/SABA) vs L ICS/SABA**					
Direct estimate	1	0.56		0.51	[0.01; 26.71]
Indirect estimate				1.62	[0.02; 142.07]
Network estimate				0.84	[0.04; 16.42]
**As-needed (ICS/SABA) vs L SMART**					
Direct estimate	1	0.62		1.19	[0.15; 9.51]
Indirect estimate				1.97	[0.14; 28.68]
Network estimate				1.44	[0.28; 7.43]
**As-needed (ICS/SABA) vs placebo**					
Direct estimate	2	0.82	0	0.63	[0.12; 3.38]
Indirect estimate				0.39	[0.01; 13.41]
Network estimate				0.57	[0.13; 2.63]
**Best Practice vs L ICS/LABA**					
Direct estimate	1	0.61		0.95	[0.18; 5.01]
Indirect estimate				1.36	[0.17; 11.10]
Network estimate				1.09	[0.30; 4.02]
**Best Practice vs L SMART**					
Direct estimate	2	0.78	0	1.36	[0.31; 5.98]
Indirect estimate				1.98	[0.12; 32.09]
Network estimate				1.48	[0.40; 5.46]
**Best Practice vs M ICS/LABA**					
Direct estimate	1	0.75		1.60	[0.34; 7.55]
Indirect estimate				0.98	[0.07; 14.35]
Network estimate				1.41	[0.37; 5.42]

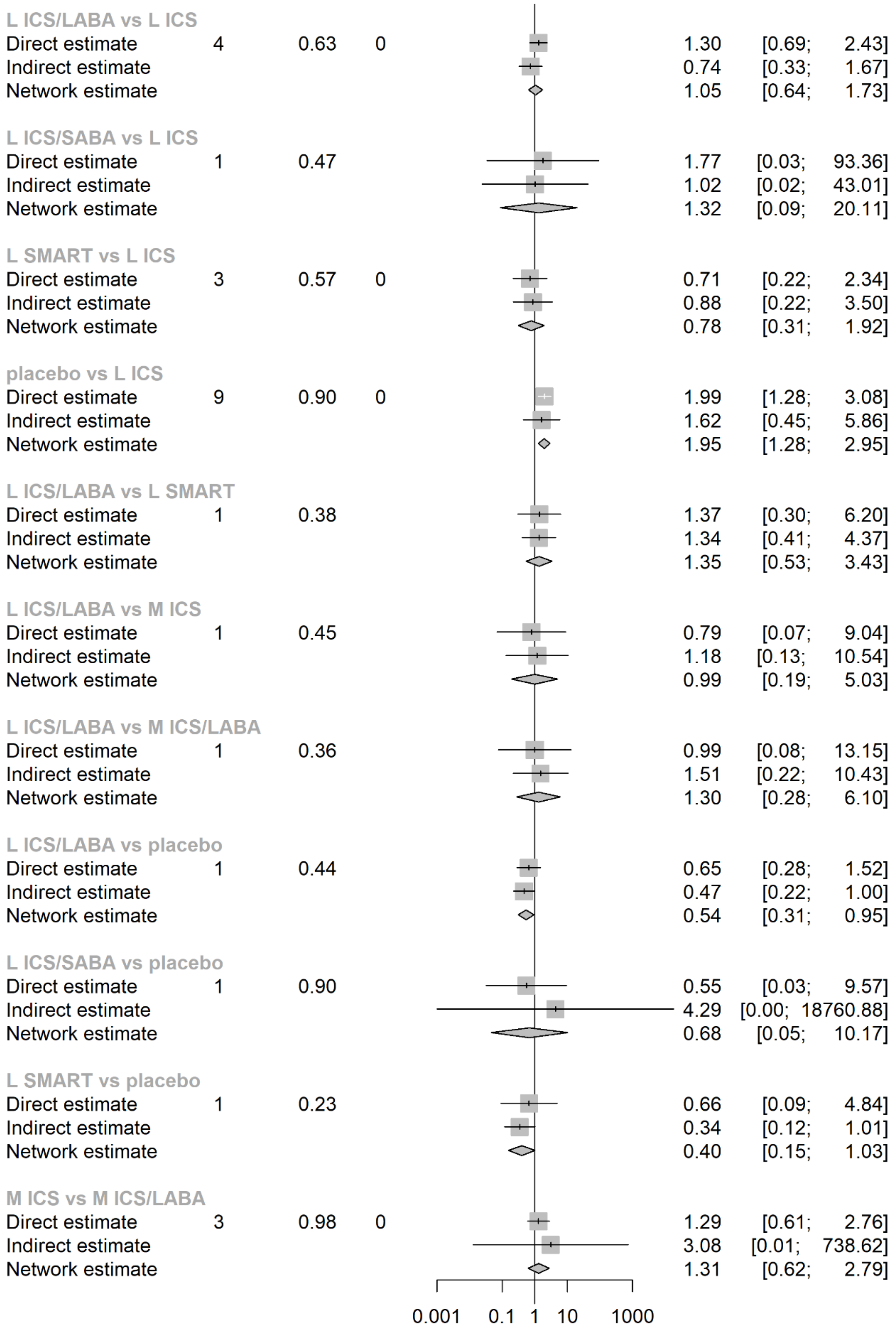

Placebo, as-needed SABA or LABA.

Supplementary Figure S3. Net heat graph of inhalers for severe exacerbation.

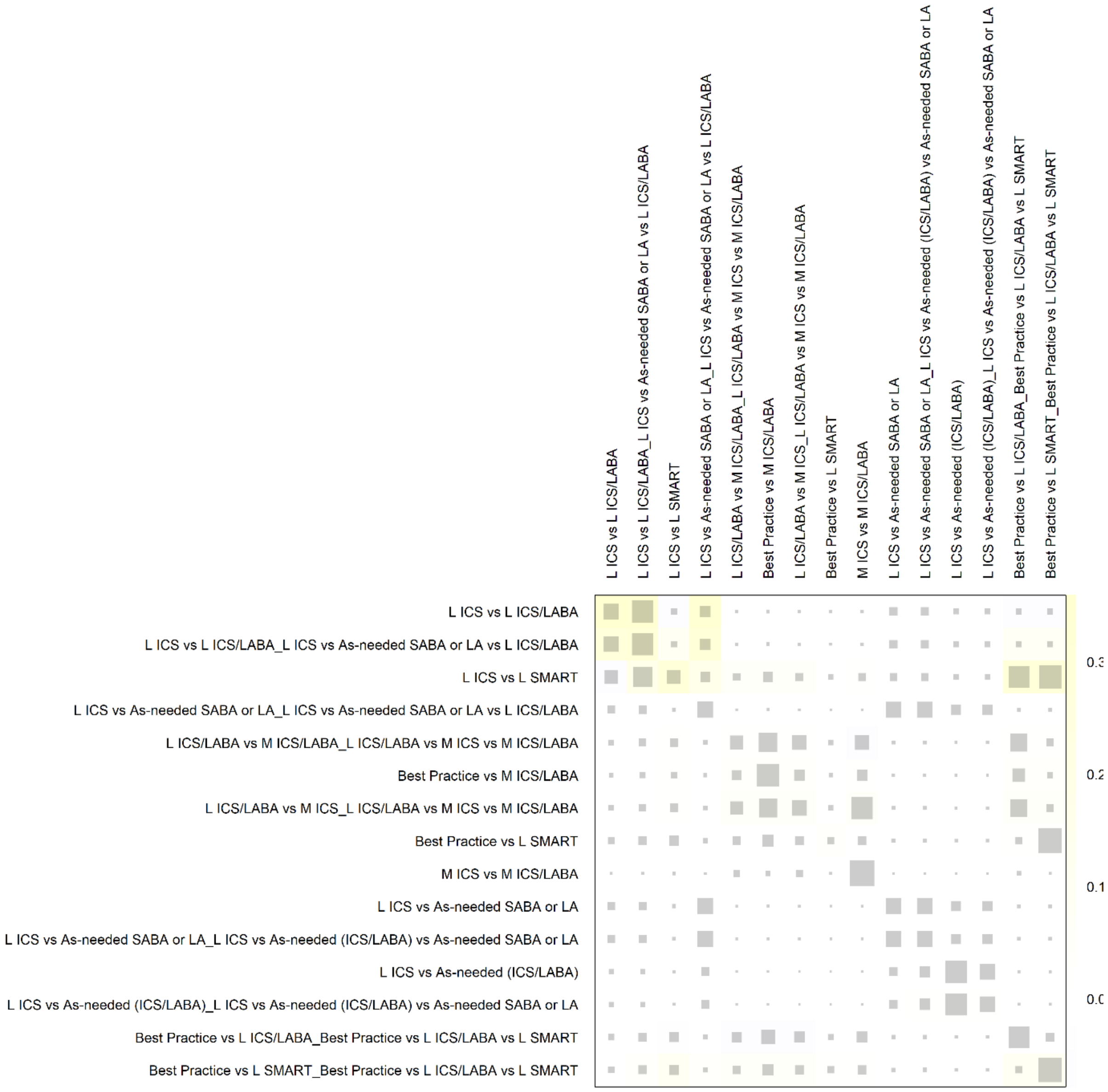

As we used the random-effects model, there was no significant inconsistency of direct and indirect comparisons among inhaler therapies.

Supplementary Figure S4. Net heat graph of inhalers for moderate-to-severe exacerbation.

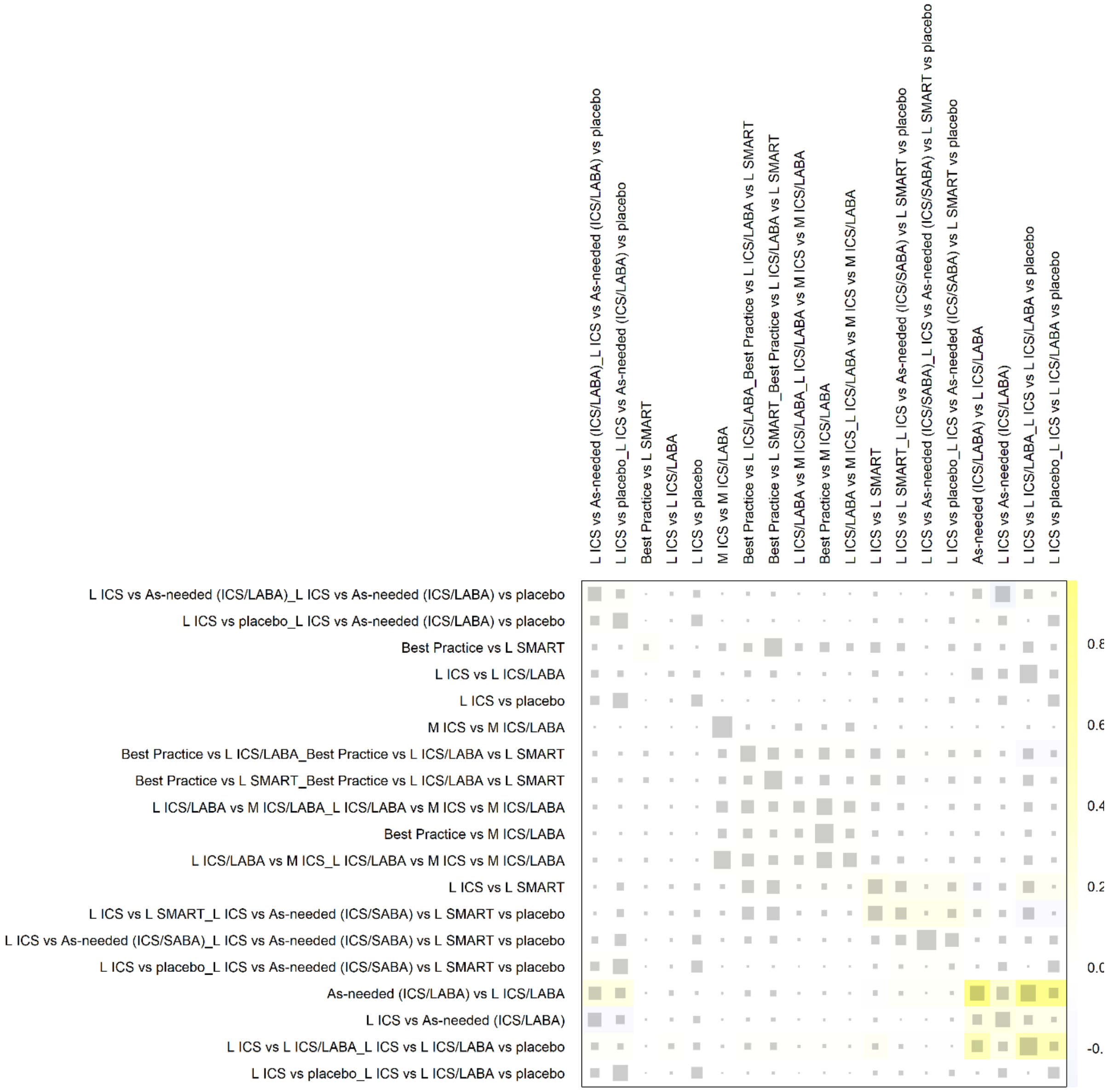

As we used the random-effects model, there was no significant inconsistency of direct and indirect comparisons among inhaler therapies. Placebo, as-needed SABA or LABA.

Supplementary Figure S5. Comparison-adjusted funnel plot of studies for moderate to severe exacerbations.

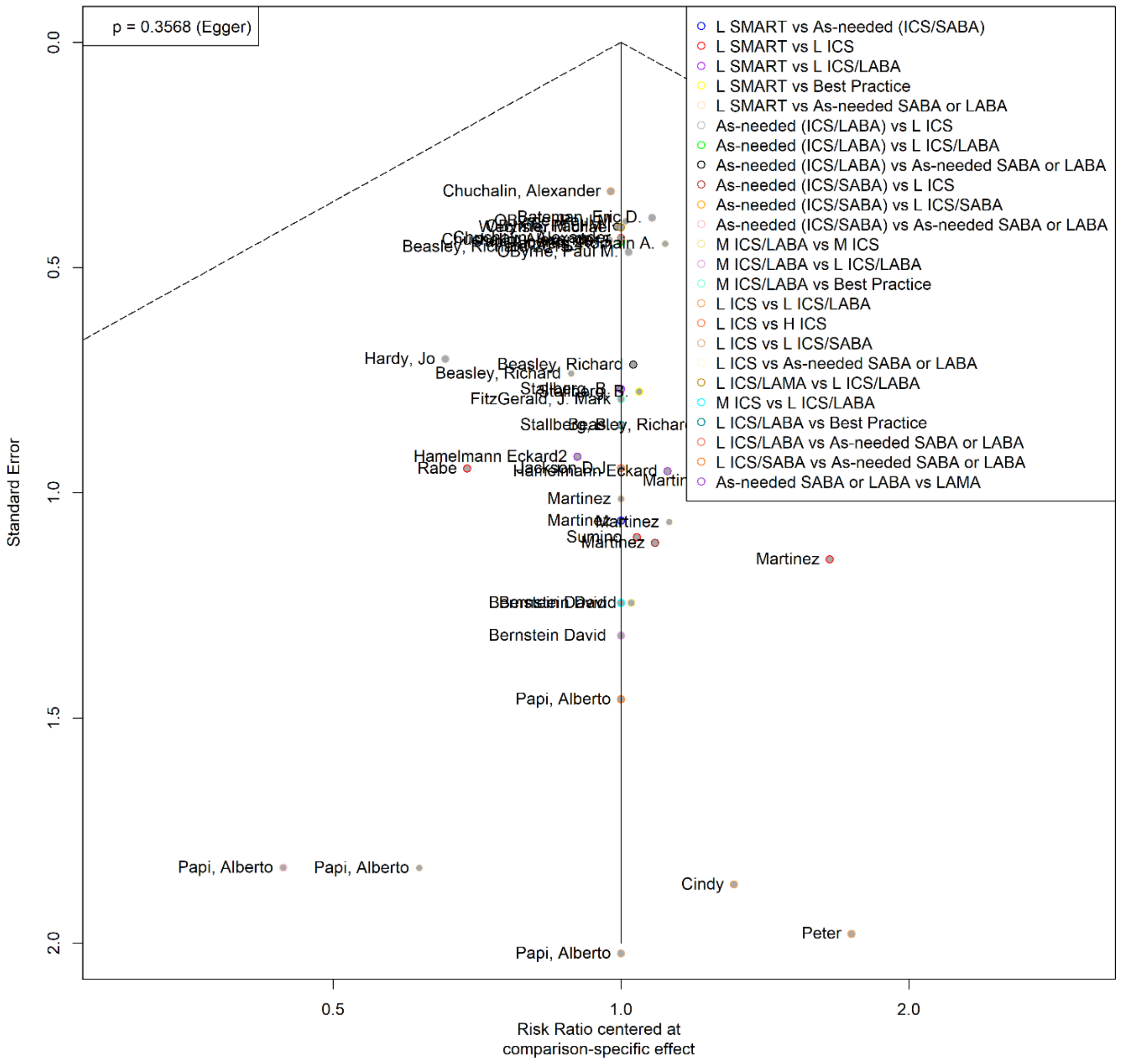

Placebo, as-needed SABA or LABA.

Supplementary Figure S6. Network of the strategies for FEV_1.

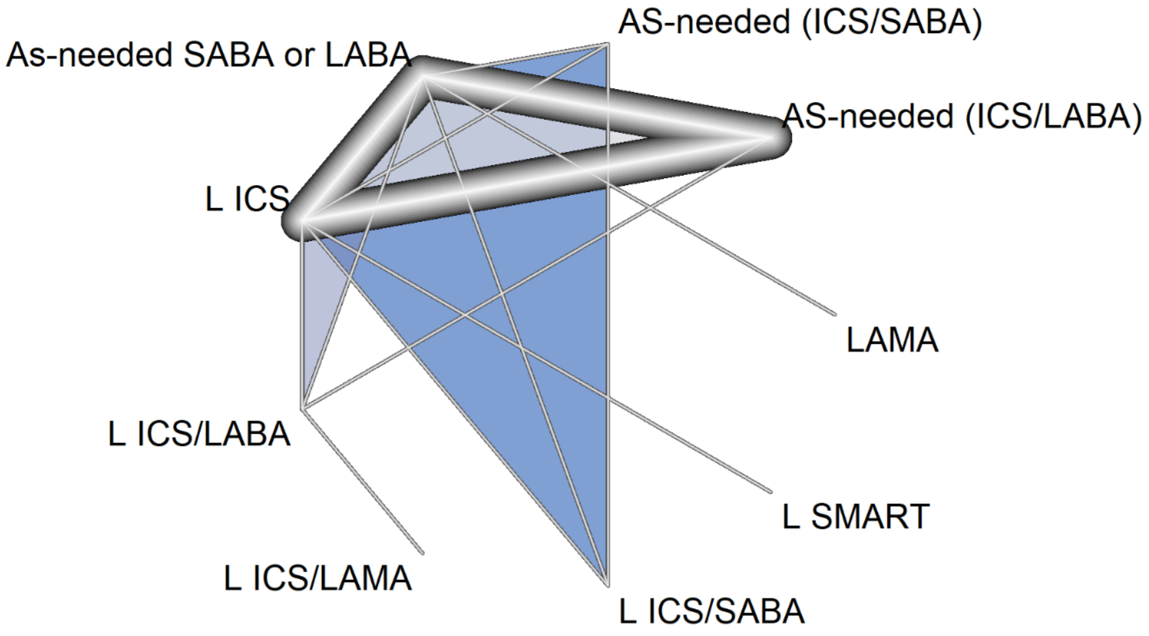

Supplementary Figure S7. Comparison of direct and indirect estimates for FEV_1 change.

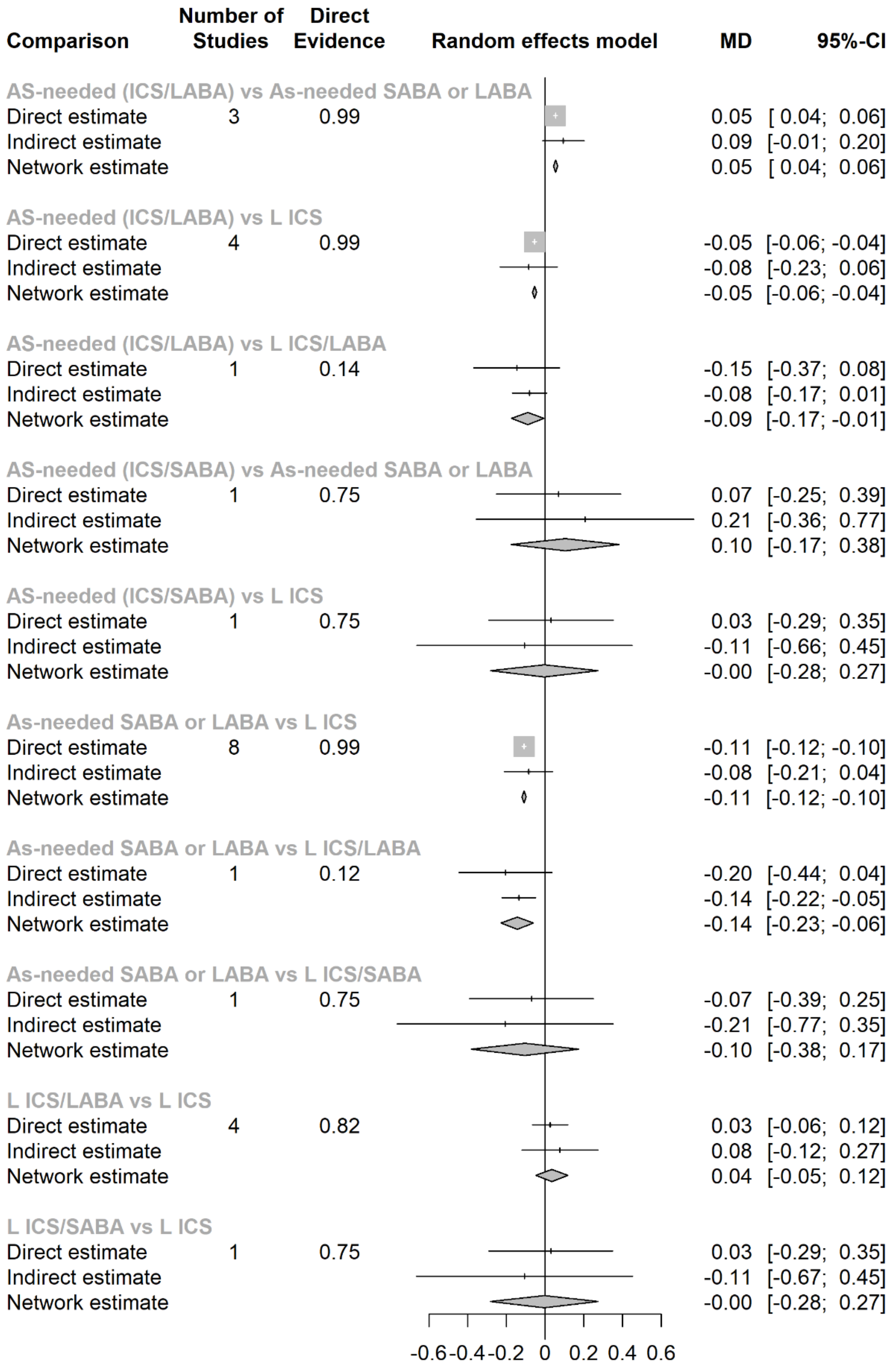

Supplementary Figure S8. Net heat graph of inhalers for FEV_1 change.

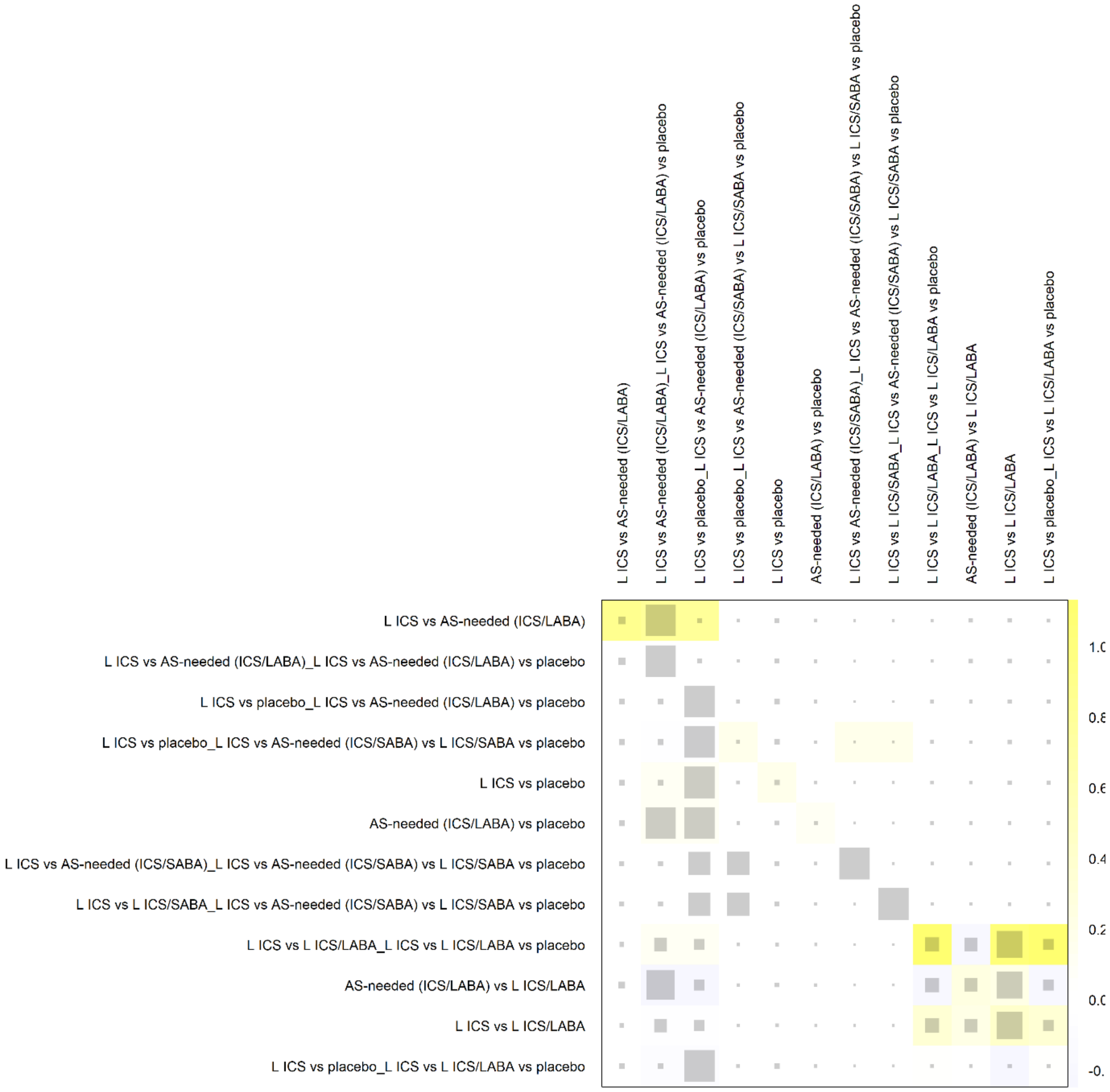

As we used the random-effects model, there was no significant inconsistency of direct and indirect comparisons among inhaler therapies. Placebo, as-needed SABA or LABA.

Supplementary Figure S9. Comparison-adjusted funnel plot of studies for FEV_1 change.

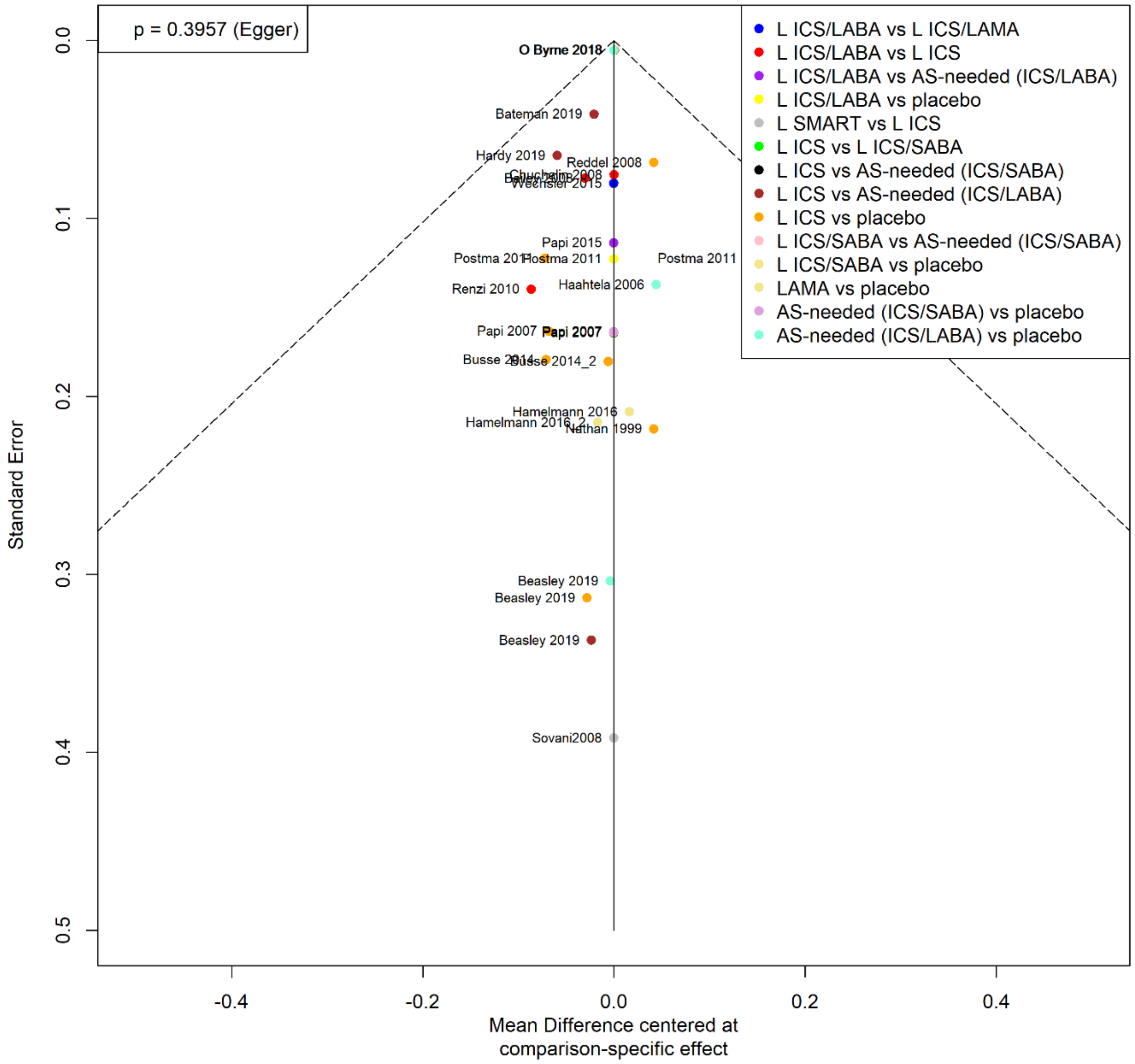

Placebo, as-needed SABA or LABA.

Supplementary Figure S10. Network of the strategies for the ACQ.

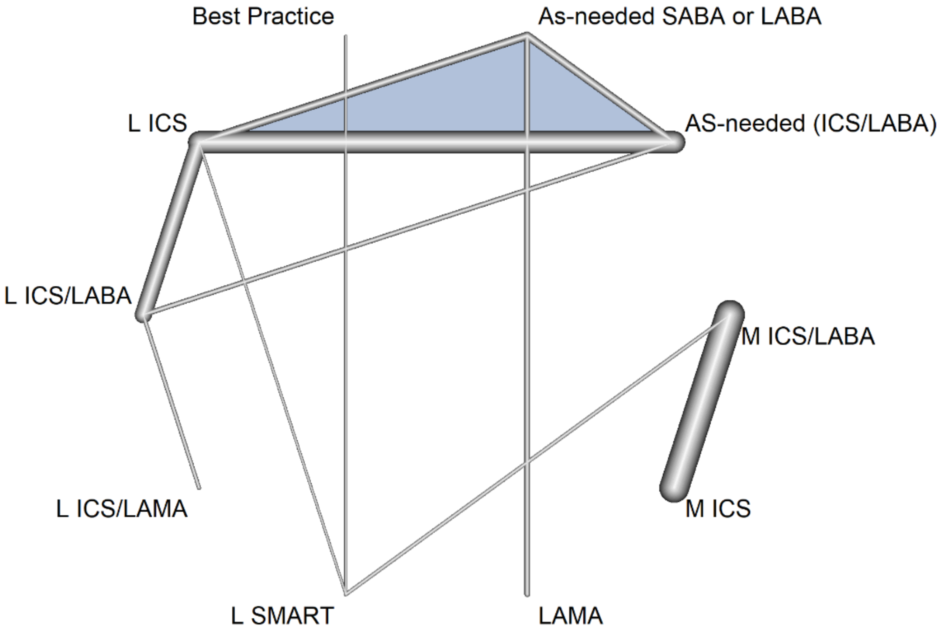

Supplementary Figure S11. Comparison of direct and indirect estimates for the ACQ.

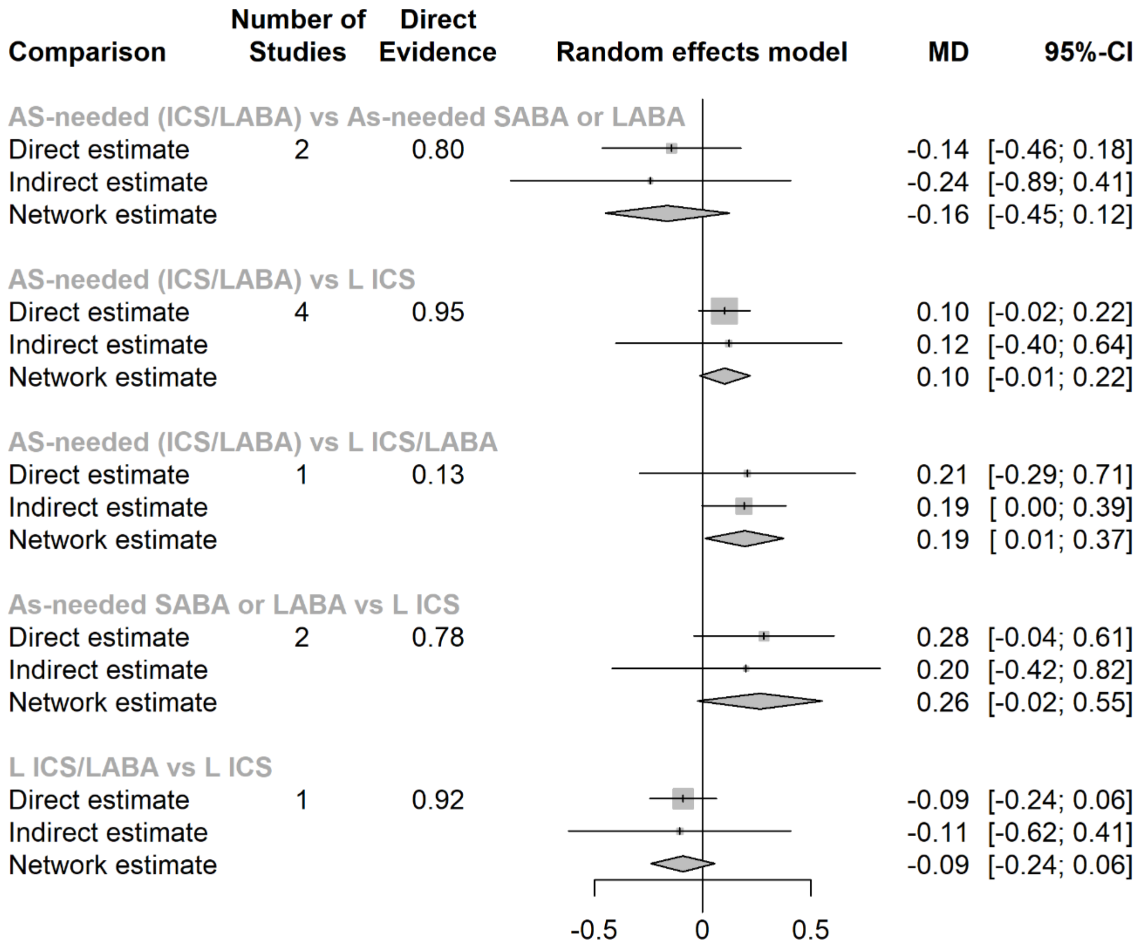

Placebo, as-needed SABA or LABA.

Supplementary Figure S12. Net heat graph of inhalers for the ACQ.

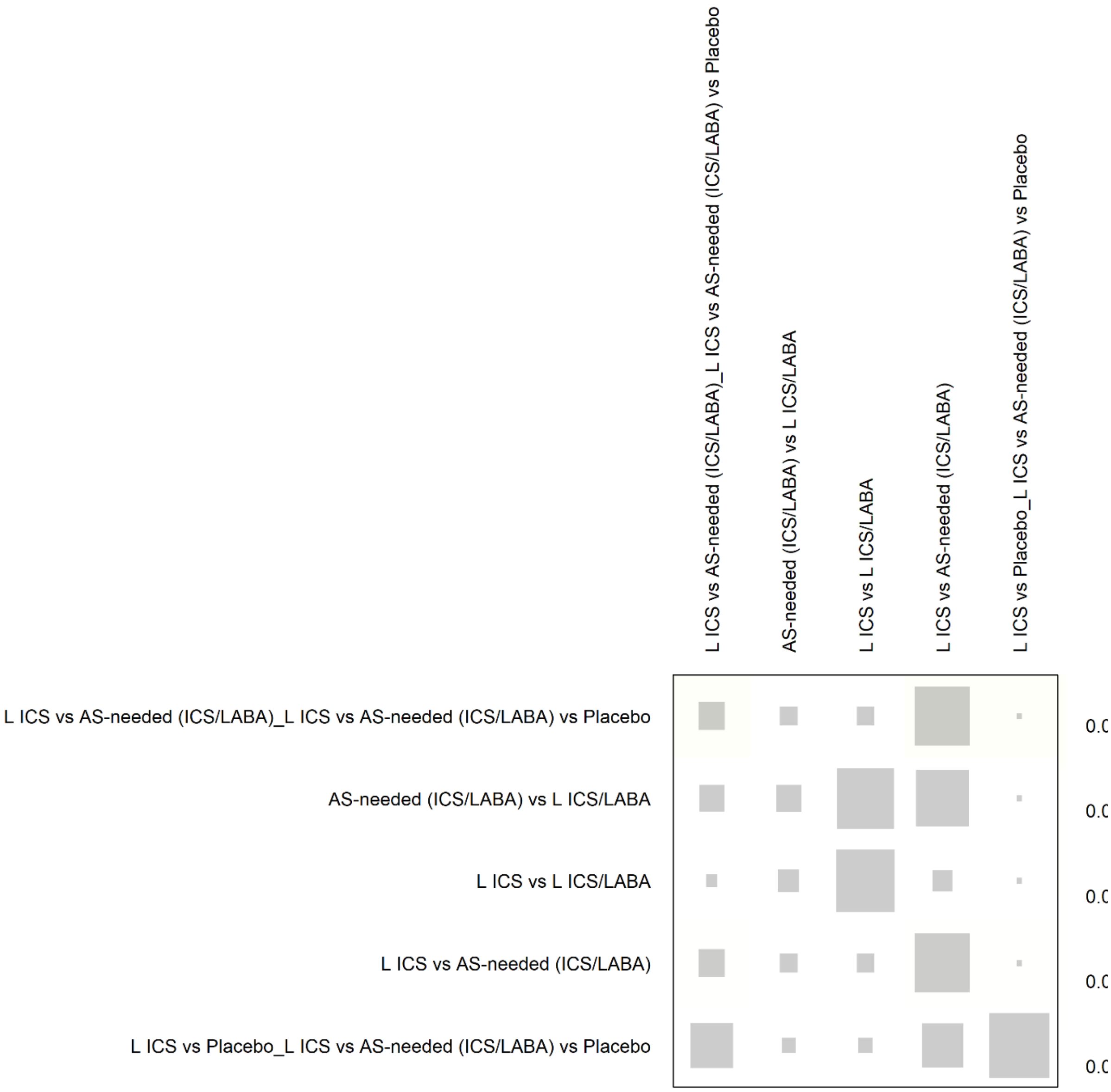

As we used the random-effects model, there was no significant inconsistency of direct and indirect comparisons among inhaler therapies.

Supplementary Figure S13. Comparison-adjusted funnel plot of the studies for the ACQ.

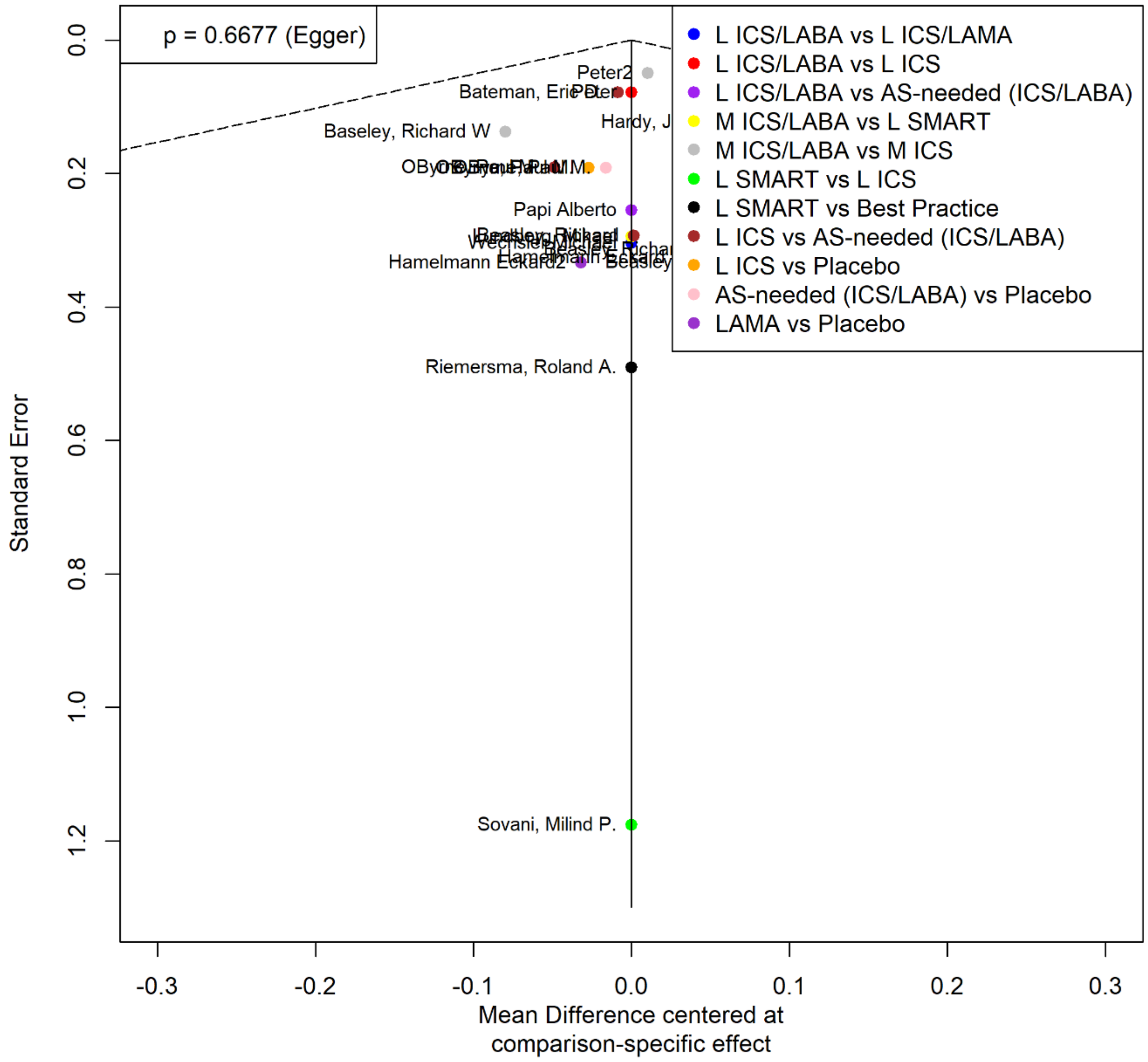

Placebo, as-needed SABA or LABA.

Supplementary Figure S14. Forest plot and network of strategies with respect to change of FEV_1 (full comparison).

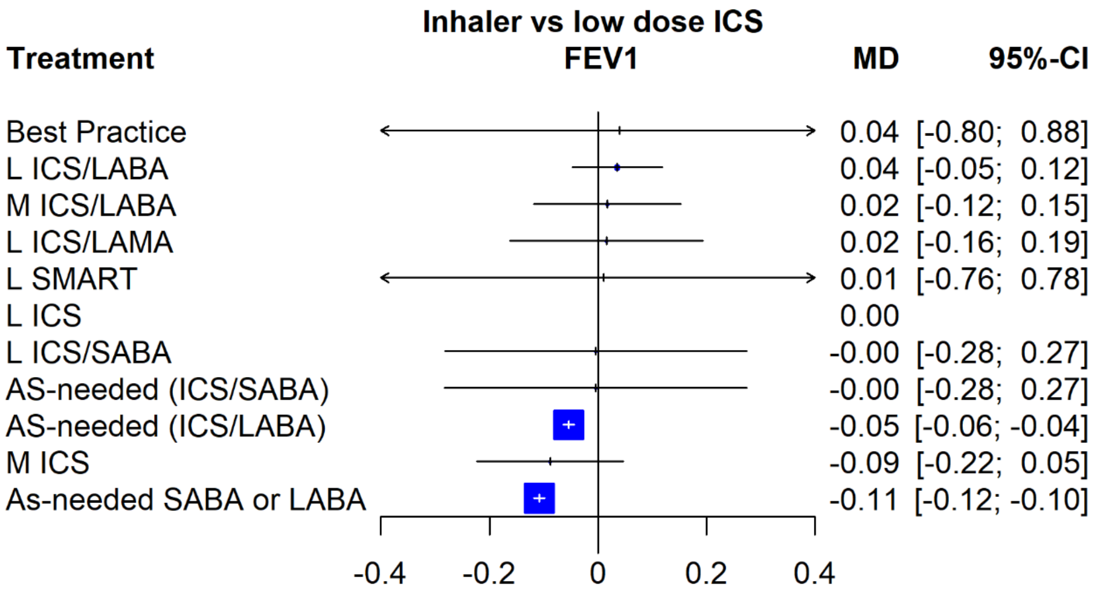

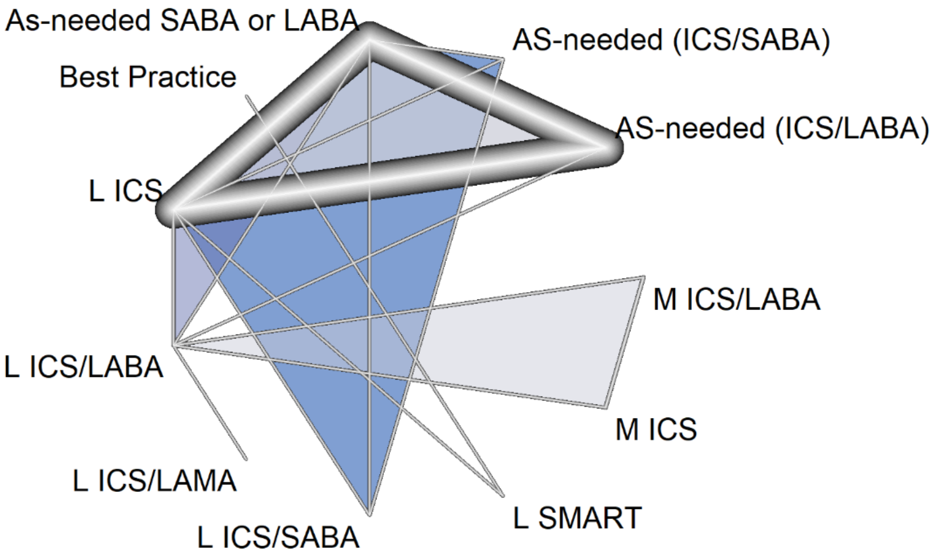

Reference

1. Reddel, H. K. *et al.* An official American Thoracic Society/European Respiratory Society statement: Asthma control and exacerbations - Standardizing endpoints for clinical asthma trials and clinical practice. *Am. J. Respir. Crit. Care Med.* **180**, 59–99 (2009).

2. Wechsler, M. E. *et al.* Anticholinergic vs long-acting β-Agonist in combination with inhaled corticosteroids in black adults with asthma: The BELT randomized clinical trial. *JAMA - J. Am. Med. Assoc.* **314**, 1720–1730 (2015).

3. Bailey, W. *et al.* Asthma exacerbations in African Americans treated for 1 year with combination fluticasone propionate and salmeterol or fluticasone propionate alone. *Curr. Med. Res. Opin.* **24**, 1669–1682 (2008).

4. Beasley, R. W. *et al.* Effect of once-daily indacaterol maleate/mometasone furoate on exacerbation risk in adolescent and adult asthma: A double-blind randomised controlled trial. *BMJ Open* **5**, (2015).

5. Bateman, E. D. *et al.* As-Needed Budesonide–Formoterol versus Maintenance Budesonide in Mild Asthma. *N. Engl. J. Med.* **378**, 1877–1887 (2018).

6. Beasley, R. *et al.* Controlled trial of budesonide-formoterol as needed for mild asthma. *N. Engl. J. Med.* **380**, 2020–2030 (2019).

7. Bernstein, D., Andersen, L., Forth, R., Jacques, L. & Yates, L. Once-daily fluticasone furoate/vilanterol versus twice-daily fluticasone propionate/salmeterol in patients with asthma well controlled on ICS/LABA. *J. Asthma* **55**, 984–993 (2018).

8. Busse, W. W. *et al.* Once-daily fluticasone furoate 50 mcg in mild-to-moderate asthma: A 24-week placebo-controlled randomized trial. *Allergy Eur. J. Allergy Clin. Immunol.* **69**, 1522–1530 (2014).

9. Chuchalin, A., Jacques, L. & Frith, L. Salmeterol/fluticasone propionate via Diskus™ once daily versus fluticasone propionate twice daily in patients with mild asthma not previously receiving maintenance corticosteroids. *Clin. Drug Investig.* **28**, 169–181 (2008).

10. FitzGerald, J. M., Boulet, L. P. & Follows, R. M. A. The CONCEPT trial: A 1-year, multicenter, randomized, double-blind, double-dummy comparison of a stable dosing regimen of salmeterol/fluticasone propionate with an adjustable maintenance dosing regimen of formoterol/ budesonide in adults with persistent a. *Clin. Ther.* **27**, 393–406 (2005).

11. Haahtela, T. *et al.* Formoterol as needed with or without budesonide in patients with intermittent asthma and raised NO levels in exhaled air: A SOMA study. *Eur. Respir. J.* **28**, 748–755 (2006).

12. Hardy, J. *et al.* Budesonide-formoterol reliever therapy versus maintenance budesonide plus terbutaline reliever therapy in adults with mild to moderate asthma (PRACTICAL): a 52-week, open-label, multicentre, superiority, randomised controlled trial. *Lancet* **394**, 919–928 (2019).

13. Jackson, D. J. *et al.* Quintupling Inhaled Glucocorticoids to Prevent Childhood Asthma Exacerbations. *Physiol. Behav.* **176**, 139–148 (2018).

14. Lundborg, M. *et al.* Maintenance plus reliever budesonide/formoterol compared with a higher maintenance dose of budesonide/formoterol plus formoterol as reliever in asthma: An efficacy and cost-effectiveness study. *Curr. Med. Res. Opin.* **22**, 809–821 (2006).

15. Nathan, R. A. *et al.* A six-month, placebo-controlled comparison of the safety and efficacy of salmeterol or beclomethasone for persistent asthma. *Ann. Allergy, Asthma Immunol.* **82**, 521–529 (1999).

16. Martinez, F. D. *et al.* Use of beclomethasone dipropionate as rescue treatment for children with mild persistent asthma (TREXA): A randomised, double-blind, placebo-controlled trial. *Lancet* **377**, 650–657 (2011).

17. O'Byrne, P. M. *et al.* Inhaled Combined Budesonide–Formoterol as Needed in Mild Asthma. *N. Engl. J. Med.* **378**, 1865–1876 (2018).

18. Papi, A. *et al.* Regular versus as-needed budesonide and formoterol combination treatment for moderate asthma: A non-inferiority, randomised, double-blind clinical trial. *Lancet Respir. Med.* **3**, 109–119 (2015).

19. Papi, A. *et al.* Rescue use of beclomethasone and albuterol in a single inhaler for mild asthma. *N. Engl. J. Med.* **356**, 2040–2052 (2007).

20. Pauwels, R. A. *et al.* Early intervention with budesonide in mild persistent asthma: A randomised, double-blind trial. *Lancet* **361**, 1071–1076 (2003).

21. Peters, S. P. *et al.* Serious asthma events with Budesonide Plus Formoterol vs. Budesonide Alone. *N. Engl. J. Med.* **375**, 850–860 (2016).

22. Postma, D. S., O'Byrne, P. M. & Pedersen, S. Comparison of the effect of low-dose ciclesonide and fixed-dose fluticasone propionate and salmeterol combination on long-term asthma control. *Chest* **139**, 311–318 (2011).

23. Rabe, K. F. *et al.* Budesonide/formoterol in a single inhaler for maintenance and relief in mild-to-moderate asthma: A randomized, double-blind trial. *Chest* **129**, 246–256 (2006).

24. Reddel, H. K., Belousova, E. G., Marks, G. B. & Jenkins, C. R. Does continuous use of inhaled corticosteroids improve outcomes in mild asthma? A double-blind randomised controlled trial. *Prim. Care Respir. J.* **17**, 39–45 (2008).

25. Renzi, P. M., Howard, L. A., Ortega, H. G., Ahmad, F. F. & Chapman, K. R. Low-dose fluticasone propionate with and without salmeterol in steroid-naïve patients with mild, uncontrolled asthma. *Respir. Med.* **104**, 510–517 (2010).

26. Riemersma, R. A., Postma, D. & van der Molen, T. Budesonide/formoterol maintenance and reliever therapy in primary care asthma management: Effects on bronchial hyperresponsiveness and asthma control. *Prim. Care Respir. J.* **21**, 50–56 (2012).

27. Sovani, M. P. *et al.* Poor adherence with inhaled corticosteroids for asthma: Can using a single inhaler containing budesonide and formoterol help? *Br. J. Gen. Pract.* **58**, 37–43 (2008).

28. Ställberg, B. *et al.* A real-life cost-effectiveness evaluation of budesonide/formoterol maintenance and reliever therapy in asthma. *Respir. Med.* **102**, 1360–1370 (2008).

29. Sumino, K. *et al.* A Pragmatic Trial of Symptom-Based Inhaled Corticosteroid Use in African-American Children with Mild Asthma. *J. Allergy Clin. Immunol. Pract.* **8**, 176-185.e2 (2020).

30. Weinstein, C. L. J. *et al.* A phase 3 study evaluating the safety and efficacy of a pediatric dose of mometasone furoate with and without formoterol for persistent asthma. *Pediatr. Pulmonol.* **55**, 882–889 (2020).
